# Transcriptome analysis revealed the role of mTOR and MAPK signaling pathways in the white strain of *Hypsizygus marmoreus* extracts-induced cell death of human hepatoma Hep3B cells

**DOI:** 10.3389/fphar.2022.1039376

**Published:** 2022-11-25

**Authors:** Kun-Tsung Lee, Li-Yun Chen, Wei-Sung Li, Hong-Zin Lee

**Affiliations:** ^1^ Department of Oral Hygiene, College of Dental Medicine, Kaohsiung Medical University, Kaohsiung, Taiwan; ^2^ Department of Dentistry, Kaohsiung Medical University Hospital, Kaohsiung Medical University, Kaohsiung, Taiwan; ^3^ School of Pharmacy, China Medical University, Taichung, Taiwan; ^4^ Plant Pathology Division, Taiwan Agricultural Research Institute, Council of Agriculture, Executive Yuan, Taichung, Taiwan

**Keywords:** *Hypsizygus marmoreus*, white genius mushroom, human Hep3B liver cancer cells, next generation sequencing technology, mTOR signaling pathway, MAPK signaling pathway, migration, cancer chemopreventive agent

## Abstract

The aim of this study was to investigate the anticancer mechanisms of white genius mushroom (WGM). WGM is a popular edible mushroom in Taiwan and has been demonstrated to mediate potent antiproliferation effects against human Hep3B liver cancer cells in our previous study. According to next generation sequencing technology and KEGG pathway enrichment analysis, mTOR and MAPK signaling pathways were markedly changed during treatment with WGM extracts in Hep3B cells. Therefore, this study examined the effects of WGM extracts on the expression of mTOR and MAPK signaling pathway-related proteins, such as PI3K, Akt, mTOR, Ras, Raf, MEK, ERK, p38 and JNK in Hep3B cells. According to the results of immunoblotting, we demonstrated that the protein expression of the members of PI3K/Akt/mTOR and MAPK signaling pathways were involved in WGM extracts-induced cell death. Furthermore, the inhibitors of PI3K/Akt/mTOR and MAPK signaling pathways such as rapamycin, MK2206, LY3214996 and SB202190, blocked the induction of cell death and vacuoles formation induced by WGM extracts. This study also demonstrated that WGM extracts is able to inhibit Hep3B cell migration and colony formation in a dose-dependent manner. In addition to being a very popular food, WGM should be a pharmacologically safe natural agent for cancer treatment. Therefore, WGM might be designed to develop into a dietary chemopreventive agent for the cancer treatment.

## Introduction

Cancer is a major public health concern and the leading cause of death in Taiwan. According to the report provided by the Ministry of Health and Welfare in Taiwan, liver cancer is always the second leading cause of cancer death for the past 20 years. Drug resistance continues to be the principal reason for achieving cures in patients with liver cancer. In recent years, an advent of cancer cells develop resistance to anticancer drugs has led researchers to expedite and put in more effort in the development of new and more effective anticancer drugs. Cancer chemoprevention is defined as the use of food supplements or synthetic compounds to suppress, prevent or delay cancer development and progression. Natural products, such as fruits and vegetables, were also receiving renewed attention as the discovery of cancer chemopreventive agents. For prevention the development of the liver cancer by blocking the initiation stage of tumorigenesis, the development of an effective cancer chemopreventive agent derived from the daily intake of food is urgently needed.

Mushrooms have been shown to have numerous biological activities including anticancer activity, antimicrobial effect and immunomodulating effects (Bao and You, 2011; Kim et al., 2011; Kwak et al., 2015). Based on the consideration of anticancer activity and immune-promoting effect, mushrooms are people’s favourite food. Anticancer activity of mushrooms against human cancer cells, such as breast, prostate and colorectal cancer cells, involves apoptosis, cell cycle arrest and inhibition of cell proliferation (Hu et al., 2002; Stanley et al., 2005; Hsu et al., 2008). Transcriptome profiling is an effective tool in large-scale investigation of gene expression patterns in distinct cellular states. Next generation sequencing (NGS) refers to massive scale RNA sequencing technology that allows investigation of a transcriptome profiling. Therefore, NGS has been widely used in the detection of changes in gene expression in different groups or treatments. KEGG (Kyoto Encyclopedia of Genes and Genomes) is a collection of databases dealing with metabolism, genetic information processing, environmental information processing, cellular processes, organismal systems, human diseases and drug development (Kanehisa and Goto, 2000). KEGG pathway enrichment facilitates to determine the differential expressed genes involved in the most important signal transduction pathways and metabolic pathways.

Phosphatidylinositol 3-kinase (PI3K)/Akt/mammalian target of rapamycin (mTOR) signaling is one of the most important intracellular signaling pathways that is related to cell proliferation, motility, survival and apoptosis of cancer cells (Katso et al., 2001; Engelman et al., 2006). Many reports have indicated that inhibition of PI3K/Akt/mTOR signaling pathway triggers apoptosis and inhibits the proliferation of tumor cells, which have elevated Akt levels (Hennessy et al., 2005; Mandal et al., 2005). Activation of the PI3K/Akt/mTOR pathway leads to tumor development and anticancer drugs resistance (Hennessy et al., 2005; Martini et al., 2014). The Ras/Raf/ERK signaling and PI3K/Akt signaling pathway are highly interconnected (Castellano and Downward, 2011). MAPK (mitogen-activated protein kinase) cascades plays a key role in the cellular response to extracellular stimuli (Raingeaud et al., 1995; Liu et al., 1996). MAPK signaling pathway has been found to be involved in the proliferation, differentiation and apoptosis of human cancers (You et al., 2011; Guo et al., 2016). Three well-defined subgroups of mammalian MAPKs are extracellular signal-regulated kinase (ERK1/2), jun N-terminal kinase/stress activated protein kinase (JNK/SAPK) and p38. Among all MAPK signal transduction pathways, the Ras/Raf/ERK signaling pathway is the most important signaling cascade and plays an important role in the proliferation and migration of tumor cells (Roberts and Der, 2007; Sun et al., 2015). It has been reported that inhibition of ERK signaling pathway can result in both decreased cellular proliferation and increased cellular death (Liu et al., 2018; Li et al., 2020).

White genius mushroom (WGM) is white strain of *H. marmoreus* and is one of the most important edible mushrooms in Taiwan. In our previous study, we have identified the cytotoxicity of white genius mushroom extracts on Hep3B cells which is partially dependent on the production of ROS in Hep3B cells (Li et al., 2022). In order to get a better understanding of the underlying mechanisms of WGM extracts-produced anticancer activity of Hep3B cells, the differential expressed genes and pathways involved in the WGM extracts-induced cell death of Hep3B cells were identified using the NGS technology and KEGG tool. According to the KEGG pathway enrichment analysis, autophagy, mitophagy and apoptosis pathways were markedly changed by WGM extracts in human Hep3B liver cancer cells (Li et al., 2022). Although WGM extracts were found to have anticancer activities, the exact mechanism of the anticancer effect of WGM extracts is substantially unknown. In this study, WGM extracts was examined for its anticancer activities and exact mechanisms in hepatocellular carcinoma Hep3B cells, and expected to develop into a dietary chemopreventive agent in the future.

## Materials and methods

### Materials

Chloroquine, MK2206, rapamycin and Temuterkib (LY3214996) were from MedChemExpress (Monmouth Junction, NJ, United States). SB202190 was purchased from Calbiochem (San Diego, CA, United States). Antibodies to various proteins were obtained from the following sources: β-Actin, Akt, Aktp (S473), JNKp(Thr183/Tyr185), MEK, p38p (Thr180/Tyr182), PI3K, Ras and mTOR were purchased from GeneTex Inc. (Irvine, CA, United States). ERK, p38 and Raf were purchased from BD Biosciences (San Diego, CA, United States). JNK was from Cell Signaling Technology (Danvers, MA, United States). ERK1p(Thr202/Tyr204)/ERK2p(Thr185/Tyr187) was from Thermo Fisher Scientific (Waltham, MA, United States). Horseradish peroxidase (HRP)-conjugated goat anti-mouse and -rabbit IgG were from Abcam (Cambridge, MA, United States).

### Preparation of white genius mushroom

White genius mushroom used in this study was harvested in September and purchased form 8329 Farm (Changhua, Taiwan). The voucher specimens of white genius mushroom (CMU-RX-HM-2020021) were deposited in School of Pharmacy, China Medical University, Taichung, Taiwan. The air-dried WGM (122.2 g) was soaked thrice with 1 L of 95% ethanol at room temperature for 3 days. The combined filtrate was then concentrated under reduced pressure at 40°C. The yield of dry extract of WGM was about 5.6%. WGM extracts are dissolved in 100% dimethylsulfoxide (DMSO) and stored at -20°C until use. In our previous study, we have demonstrated that the IC_50_ (half maximal inhibitory concentration) of WGM extracts was about 175 μg/ml (Li et al., 2022). Since the concentration of IC_50_ of WGM was used to treat the cells in the inhibitor experiment, inhibitors had no significant protective effect on the WGM-induced vacuoles formation and cell death. Therefore, IC_40_, which was about 150 μg/ml WGM, was chosen in this study.

### Cell culture

Hepatocellular carcinoma Hep3B, hepatocellular carcinoma HepG2 and lung squamous carcinoma CH27 cells were cultured in Dulbecco’s Modified Eagle Medium (Gibco BRL, Gaithersburg, MD, United States) supplemented with 10% fetal bovine serum (HyClone, Logan, UT, United States), 100 U/ml penicillin, 100 μg/ml streptomycin and 2 mM glutamine at 37°C in a humidified atmosphere with 5% CO_2_. Breast cancer MCF-7 and prostatic adenocarcinoma PC-3 cells were cultured in Minimum Essential Medium (Gibco BRL, Gaithersburg, MD, United States) and Roswell Park Memorial Institute 1640 Medium (Gibco BRL, Gaithersburg, MD, United States), respectively, supplemented with 10% fetal bovine serum, 100 U/ml penicillin, 100 μg/ml streptomycin and 2 mM glutamine. Hep3B, HepG2, MCF-7 and PC-3 cancer cell lines were purchased from the Food Industry Research and Development Institute (Hsinchu, Taiwan). CH27 cells was kindly provided by Professor Shih-Lan Hsu (Taichung Veterans General Hospital, Taichung, Taiwan).

### Total RNA extraction

Total RNA was extracted from Hep3B cells using AllPure Total RNA Isolation Kit (AllBio Science Inc., Taiwan) following the manufacturer’s protocol. RNA concentration was detected using a spectrophotometer with wavelength at 260 nm.

### Differential expressed genes analysis

Gene expression is measured using read density, with higher read density indicating higher gene expression levels. Gene expression was calculated using Cuffdiff (v2.2.1) software that calculates FPKM (fragments per kilo bases per million reads). The formula is:

$$FPKM=\frac{total\;exon\;fragments}{mapped\;read\;\left(Millions\right)\times exon\;length\;\left(KB\right)}$$

Comparison of the expression levels of all genes under different experimental conditions by FPKM profiling. Gene differential analysis was performed using the Cuffdiff (v2.2.1). Based on the criteria of fold change greater than 2 and *p*-value less than 0.05, the results of the Cuffdiff analysis were further analyzed to identify genes with significantly differential expression. Differential expressed genes (DEGs) of WGM extracts-treated samples compared to controls were analyzed using DESeq (v1.18.0) Bioconductor package, which is a model based on a negative binomial distribution. After adjustment by the Benjamini–Hochberg method for controlling the false discovery rate, *p*-values for genes were set at < 0.05 to detect differential expressed genes. The raw sequencing data were uploaded to the NCBI Sequence Read Archive (SRA) with the accession ID PRJNA813700.

### Cluster analysis of differential expressed genes

Cluster analysis is the calculation and classification of data based on similarity, thereby grouping samples or genes with similar expression patterns into one group. This can predict the function of unknown genes and predict whether they are involved in the same cellular pathway or metabolic process. The FPKM value of different genes under different experimental conditions was taken as the expression level and used for hierarchical clustering. The most obvious feature of this method is the generation of dendrogram. Different clusters are represented by different colored regions. Gene expression patterns are similar within the same cluster and close to each other, and they may have similar biological functions. The gplots in R software were used for cluster analysis, and the data of union_for_cluster were clustered by the log relative expression value of genes. We used algorithms to obtain the distance between genes, and then calculated the relative distance between the genes through repeated operations. Finally, clustering was performed by dividing genes into different subclusters based on their relative distances.

### Kyoto encyclopedia of genes and genomes enrichment analysis of differential expressed genes

KEGG is a collection of databases dealing with metabolism, genetic information processing, environmental information processing, cellular processes, organismal systems, human diseases and drug development (http://en.wikipedia.org/wiki/KEGG). We used scripts in house to enrich significant differential expressed genes in KEGG pathways. KEGG pathway units and a hypergeometric test were used to perform the pathway enrichment analysis and find the pathways of the differentially express genes that are significantly enriched in the transcriptome data. Below is the formula:

$$p=1-\overset{m-1}{\underset{i=0}{\sum }}\frac{\left(\begin{array}{c} M\\ i \end{array}\right)\left(\begin{array}{c} N-M\\ n-i \end{array}\right)}{\left(\begin{array}{c} N\\ n \end{array}\right)}$$

*N* is the number of genes with pathway annotations, n is the number of differential expressed genes in *N*, *M* is the number of genes annotated for a particular pathway in all genes, and m is the number of differential expressed genes annotated for that pathway. The threshold Q value is ≤ 0.05.

### Assessment of morphological changes

Cells were seeded at a density of 5 × 10^4^ cells per well onto 12-well plate 48 h before being treated with drugs. Hep3B cells were incubated without or with indicated various concentrations WGM extracts for 24 h. The morphology of the cells was photographed with an Olympus IX 73 phase contrast microscope at objective ×10 magnification. A field was chosen in the center of each well at approximately the same location for photography.

### Mitochondrial reductase activity assay

Mitochondrial reductase activity assay was performed as previously described (Li et al., 2022). The assay is based on the measurement of the reduction of 3-(4,5-dimethylthiazol-2-yl)-2,5-diphenyltetrazolium bromide (MTT) after white genius mushroom extracts treatment.

### Protein preparation and western blot analysis

Protein preparation and Western blot analysis were performed as previously described (Lee et al., 2009). The primary antibodies used in this study were as follows. β-Actin, 1:5000; Akt, 1:2000; Aktp (S473), 1:1500; ERK, 1:1000; ERK1p(Thr202/Tyr204)/ERK2p(Thr185/Tyr187), 1:1000; JNK, 1:2000; JNKp(Thr183/Tyr185), 1:1000; MEK, 1:2000; p38, 1:1000; p38p (Thr180/Tyr182), 1:1000; PI3K, 1:3000; Raf, 1:1000; Ras, 1:1000; mTOR, 1:1000. β-Actin was used as an internal control. The secondary antibody was used at a dilution of 1:20,000 of HRP-conjugated goat anti-mouse IgG (for β-actin, ERK, p38 and Raf) or HRP-conjugated goat anti-rabbit IgG (for Akt, Aktp (S473), ERK1p(Thr202/Tyr204)/ERK2p(Thr185/Tyr187), JNK, JNKp(Thr183/Tyr185), MEK, p38p (Thr180/Tyr182), PI3K, Ras and mTOR). The Western blot results of protein were quantified by Lane 1D Gel imaging analysis software (Sagecreation, Beijing, China).

### Wound healing assay

The migratory activity of Hep3B cells was assessed using a wounded migration assay. Wounded migration assay was performed as previously described (Liao et al., 2015). As cell reached confluence or near confluence, a linear wound was scratched across each well by a sterile 200 μl pipette tip. After washing, cells were treated with vehicle alone (control) or with 75, 125 or 150 μg/ml WGM extracts for 24 h. Images of wounds at 0 h and 24 h after scratching were obtained with an Olympus IX 73 phase-contrast microscope (Olympus Optical Co., Tokyo, Japan) at objective ×10 magnification.

### Transwell migration experiments

*In vitro* cell migration was also performed using 24-well transwell inserts with a pore size of 8 μm (Corning Life Sciences, NY, United States). The transwell chambers were used according to the manufacturer’s protocol. The cell pellets were resuspended in DMEM supplemented with 0.1% FBS at a cell density of 1 × 10^6^ cells/ml. Aliquots of 100 μl cell suspension (1 × 10^5^ cells per well) containing DMSO or WGM extracts were loaded into transwell inserts that were subsequently placed into the 24-well plate, and 600 μl medium supplemented with 10% FBS was used as a chemoattractant in the lower chamber. After incubated for 24 h at 37°C, the cells remaining on the upper surface of the membrane were removed with a cotton swab, and the cells on the lower surface of the membrane are the migrated cells. Filters were fixed with 4% paraformaldehyde solution for 20 min and stained with 0.1% crystal violet for 10 min, then the cells that passed through the filter were photographed by Olympus IX 73 phase-contrast microscope (Olympus Optical Co., Tokyo, Japan) at objective ×20 magnification.

### Colony formation assay

To assess the effects of WGM extracts on cell proliferation, the colony formation assay was carried out *in vitro*. Hep3B cells were seeded at a density of 5 × 10^3^ cells per well onto 6-well plate 48 h before drug treatment. Cells were treated with vehicle alone or with 125 or 150 μg/ml WGM extracts for 24 h. After treatment, the media was replaced with fresh complete growth media without the WGM extracts. Treated cells were washed by phosphate-buffered saline and then cultured in culture medium for 3 and 4 days with medium replacement every 2 days. After incubated at 37°C for 3 and 4 days, colonies were fixed with 4% paraformaldehyde solution and then stained with 0.5% crystal violet. Finally, the colonies were photographed.

### Data analysis and statistics

One-way ANOVA and Bonferroni post-hoc test were used to analyze differences between each experimental group. A *p*-value less than 0.05 was considered significant.

## Results

### Processing of next generation sequencing data and differential expressed genes analysis

**FIGURE 1:**
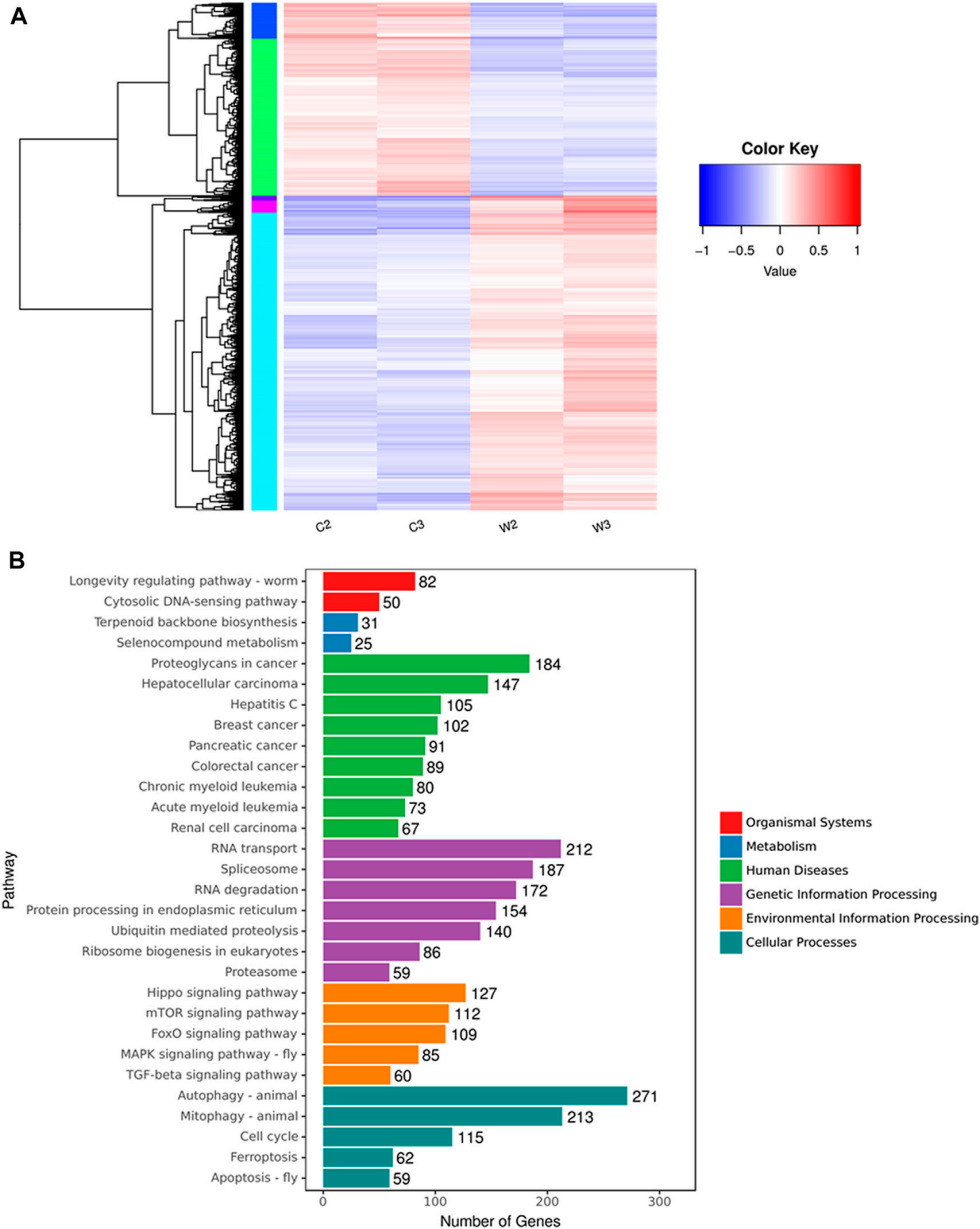
Cluster analysis and KEGG pathway enrichment of differential expressed genes between WGM extracts-treated and control groups in Hep3B cells. Cells were treated with vehicle alone or with 150 μg/ml WGM extracts for 24 h. **(A)** Cluster analysis of differential expressed genes between the WGM extracts-treated and control groups. The regions of different colors represent different clusters. The color scale of the heatmap illustrates the log_2_ of fold change of the WGM extracts-treated/control samples shown in the heatmap. **(B)** The KEGG pathway enrichments of the DEGs between WGM extracts-treated and control samples. The *y-axis* shows the names of the enriched pathways. The results were analyzed to determine genes with significant differential expression according to the criteria of fold change greater than 2 and FDR less than 0.05.

Since the exact mechanism of the anticancer effect of WGM extracts is substantially unknown, this study takes advantage of NGS technology to compare differences in the transcriptome profiles between control and WGM extracts-treated cells. The software of Cuffdiff (v2.2.1) and HTSEQ (v0.6.1) were used for gene expression calculation. Based on the criteria of fold change greater than 2 and *p*-value less than 0.05, the results of the Cuffdiff analysis were further analyzed to identify genes with significantly differential expression. To directly assess differential expressed genes (DEGs) expression in these samples and confirm the classification, a DEG heat map was drawn using the R package pheatmap (version 1.0.8). Gene expression patterns are similar within the same cluster and close to each other, and they may have similar biological functions. Cluster analysis of differentially expressed genes log_10_ (FPKM +1) values are used for clustering. Genes of low expression are in blue, and high expressed in red. The heat map clearly indicated the distinct separation of the WGM extracts-treated and control groups (Figure 1A).

### mTOR and mitogen-activated protein kinase signaling pathways were involved in white genius mushroom extracts-induced cell death of Hep3B cells

**FIGURE 2:**
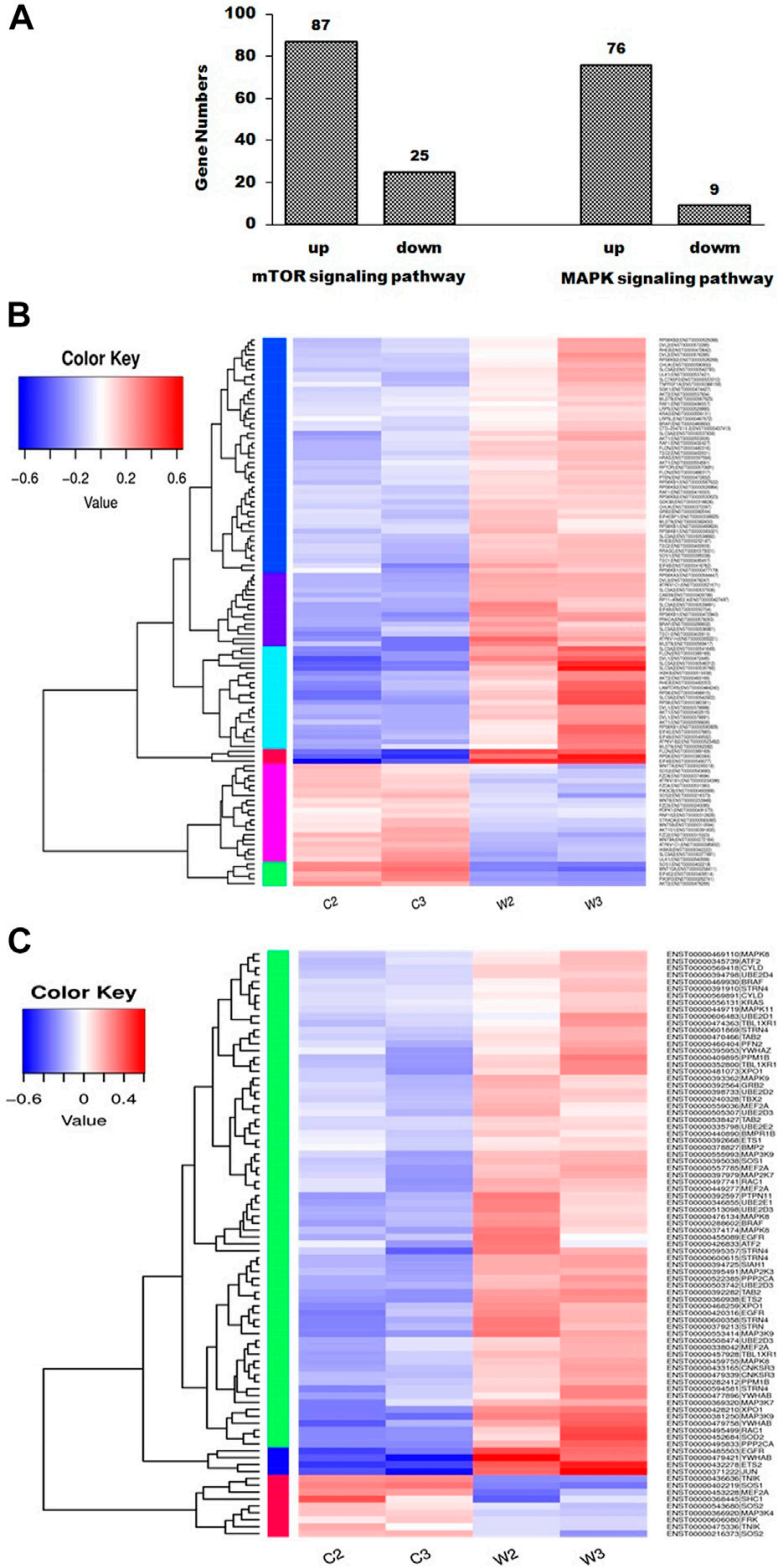
mTOR and MAPK signaling pathways were involved in WGM extracts-induced cell death of Hep3B cells. Cells were treated with vehicle alone or with 150 μg/ml WGM extracts for 24 h. **(A)** Summary of gene numbers of mTOR and MAPK signaling pathways that are significantly upregulated or downregulated between WGM extracts-treated and control cells. Differential expressed genes between WGM extracts-treated and control cells were selected based on more than 2-fold expression changes and the *p*-value was <0.05. **(B)** Cluster analysis of DEGs in mTOR signaling pathway of the WGM extracts-treated and control groups. The regions of different colors represent different clusters. The color scale of the heatmap illustrates the log_2_ of fold change of the WGM extracts-treated/control samples shown in the heatmap. **(C)** Heatmap displaying hierarchical clustering of DEG involved in MAPK signaling pathway. The regions of different colors represent different clusters. The color scale of the heatmap illustrates the log_2_ of fold change of the WGM extracts-treated/control samples shown in the heatmap.

After screening differential expressed genes, KEGG pathway enrichment analysis was subsequently performed to investigate the function of the identified genes. Pathway functional enrichment facilitates to determine the DEGs involved in the most important signal transduction pathways and metabolic pathways. The top 30 KEGG pathways are presented in Figure 1B. KEGG pathway analysis showed that the DEGs induced by WGM extracts were found to be involved in molecular pathways for organismal systems, metabolism, human diseases, genetic information processing, environmental information processing and cellular processes (Figure 1B). In the cellular processes of KEGG pathway analysis, autophagy, mitophagy and apoptosis pathways were found to be significantly changed in WGM extracts-treated cells (Figure 1B). However, the involvement of autophagy, mitophagy and apoptosis pathways in WGM extracts-induced cell death was investigated in our previously study. Hippo, mTOR, FoxO, MAPK and TGF-beta signaling pathways were significantly altered in the environmental information processing (Figure 1B). The mTOR and MAPK signaling pathways are necessary, however, to promote growth and proliferation in many mammalian cell types. We focused our attention on the investigation of the effects of WGM extracts on the expression of mTOR and MAPK signaling pathways in Hep3B cells in this study. As shown in Figure 2A, 112 genes (87 upregulated and 25 downregulated) were related to mTOR signaling pathway and 85 genes (76 upregulated and 9 downregulated) to MAPK signaling pathway. The differential expressed genes involved in mTOR and MAPK signaling pathways were shown in Supplementary Tables S1, S2, respectively. The results of a hierarchical clustering analysis of DEG involved in mTOR and MAPK signaling pathways are displayed in a heat map as a dendrogram (Figures 2B,C).

### The effects of white genius mushroom extracts on the expression of mTOR and mitogen-activated protein kinase signaling pathways-related proteins

**FIGURE 3:**
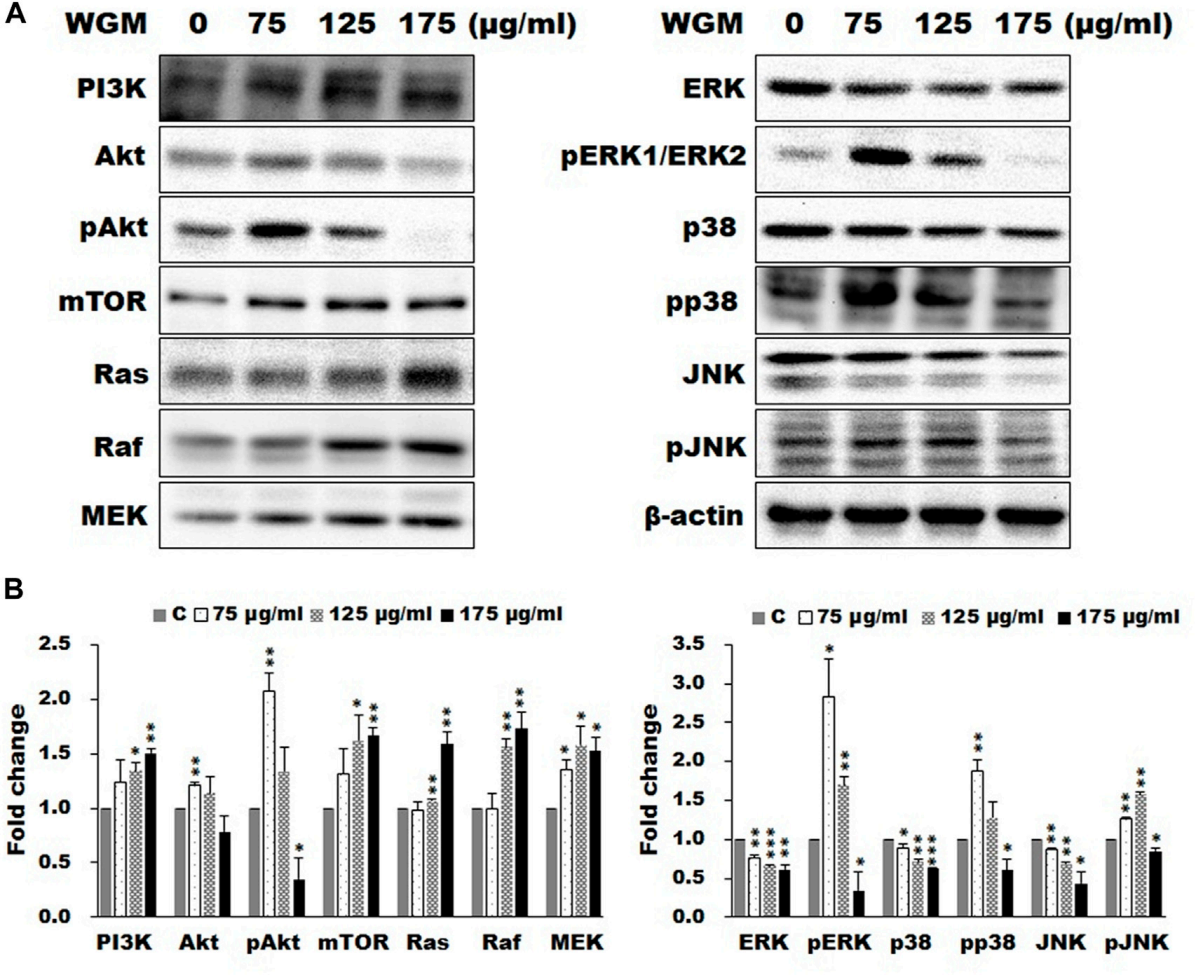
Effects of WGM extracts on the protein levels of the members of mTOR and MAP signaling pathways in Hep3B cells. The effects of WGM extracts on mTOR and MAPK signaling pathway-related proteins were analyzed by Western blotting. Cells were treated with vehicle alone or with 75, 125 or 175 μg/ml WGM extracts for 24 h. Protein samples were analyzed by SDS-PAGE (5% for PI3K and mTOR, 8% for Raf, 10% for Akt and Aktp (S473), 12% for β-actin, ERK, ERK1p(Thr202/Tyr204)/ERK2p(Thr185/Tyr187), JNK, JNKp(Thr183/Tyr185), MEK, p38 and p38p (Thr180/Tyr182) and 14% for Ras), and then probed with primary antibodies followed by secondary antibodies. **(A)** Representative blots. **(B)** The blots were quantified by Lane 1D Gel imaging analysis software. Protein expression was normalized using β-actin. Fold change = normalized signal treated/normalized signal control. Results are expressed as the mean ± S.D. of three independent experiments. *p < 0.05, **p < 0.01, ***p < 0.001 compared to the control values.

According to the KEGG pathway enrichment analysis, the present study examined the effects of WGM extracts on the expression of mTOR and MAPK signaling pathway-related proteins, such as PI3K, Akt, mTOR, Ras, Raf, MEK, ERK, p38 and JNK, in Hep3B cells. The expression of mTOR and MAPK signaling pathway-related proteins during WGM extracts-induced cell death was performed by Western blotting techniques. After treating the Hep3B cells with WGM extracts for 24 h, the protein levels of PI3K and mTOR were increased after treatment with WGM extracts for 24 h (Figure 3A). Cells treated for 24 h with WGM extracts showed a marked dose-dependent decrease in the expression of the ERK and JNK protein, and an upregulation of the Ras, Raf and MEK protein (Figure 3A). The present study also investigated the expression of pERK protein in WGM extracts-induced Hep3B cell death. Treatment with WGM extracts increased pERK protein in Hep3B cells up to 125 μg/ml, but pERK level was decreased after treatment with 175 μg/ml WGM extracts. It is interesting to note that WGM extracts induced a significant increase in the protein expression of Ras, but did not induce ERK activity, in cells exposed to 175 μg/ml WGM extracts for 24 h (Figure 3A). These results indicate that WGM extracts-mediated ERK activation occurs through a Ras-independent pathway. As shown in Figure 3A, the present study also demonstrated that the expression patterns of pERK protein are similar to those seen in Akt and pAkt expression after treatment with WGM extracts for 24 h (Figure 3A). In our study, not only the expression of ERK, which is associated with cell proliferation, but also p38, which is regulated by various environmental stresses, is regulated by WGM extracts. As shown by immunoblotting, WGM extracts caused a marked increase in the protein levels of pp38 in Hep3B cells (Figure 3A). However, the protein levels of p38 were decreased after treatment with WGM extracts (Figure 3A). As shown in Figure 3B, the Western blot results of protein were quantified by Lane 1D Gel imaging analysis software. These results indicate that changes in the expression of PI3K/mTOR and MAPK signaling pathways-related proteins in WGM extracts-treated cells is associated with WGM extracts-induced Hep3B cell death.

### The effects of the inhibitors of PI3K/Akt/mTOR signaling pathway on white genius mushroom extracts-induced cell death and membrane-enclosed vacuoles of Hep3B cells

**FIGURE 4:**
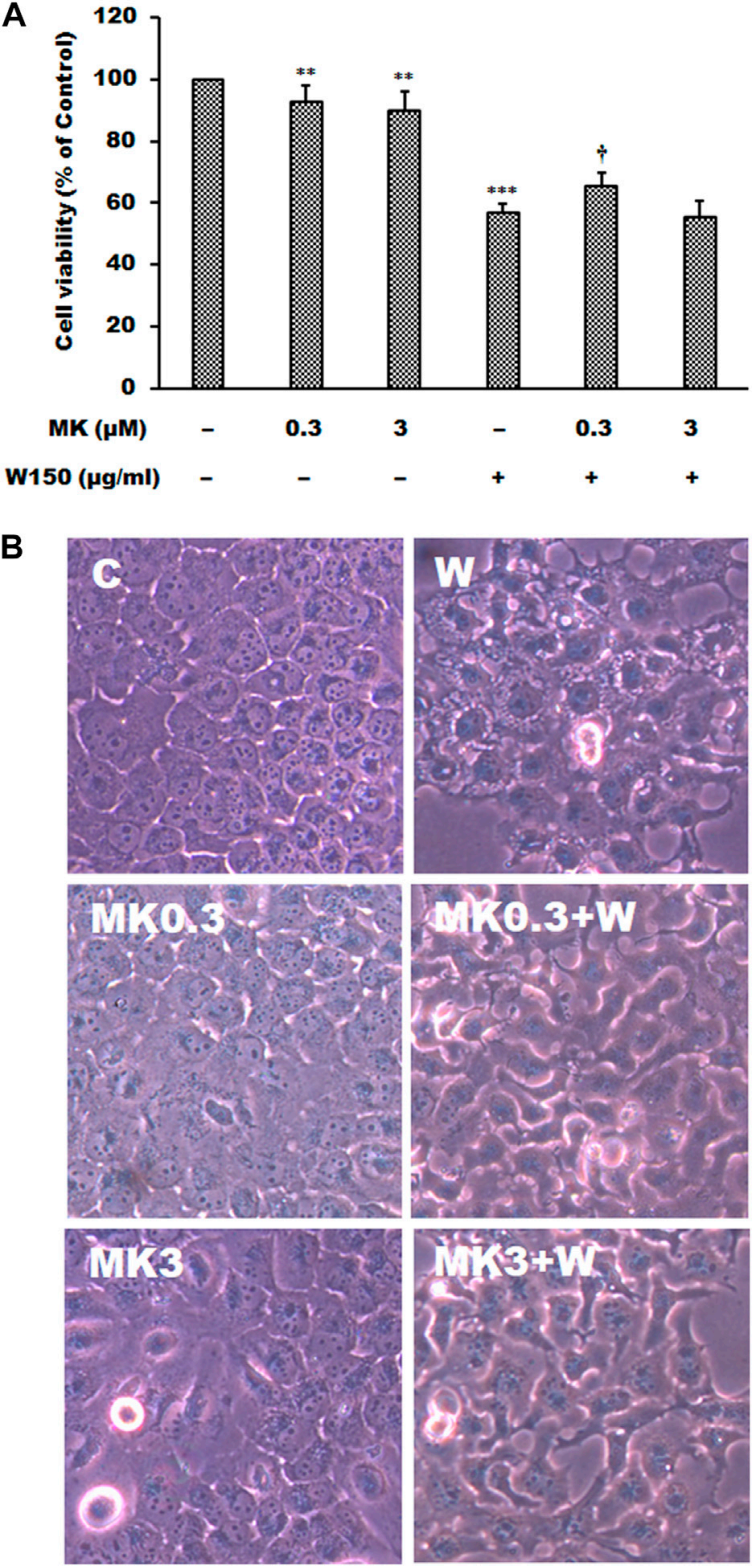
The effects of MK2206 on WGM extracts-induced cell death and membrane-enclosed vacuoles of Hep3B cells. Cells were pretreated with 0.3 or 3 μM MK2206 (MK) for 1 h and then treated with 0.1% DMSO or 150 μg/ml WGM extracts (W) for 24 h. **(A)** The effects of MK2206 on WGM extracts-induced cell death of Hep3B cells. After treatment, the viable cells were measured by MTT assay. All determinations are expressed as the mean % control ±S.D. of duplicate from three independent experiments. **p < 0.01, ***p < 0.001 compared to the control values. ^†^ *p* < 0.05 compared to the WGM extracts alone. **(B)** The effects of MK2206 on WGM extracts-induced membrane-enclosed vacuoles of Hep3B cells. After treatment, the cells were immediately photographed with an Olympus IX 73 phase-contrast microscope. All results are representative of three independent experiments.

**FIGURE 5:**
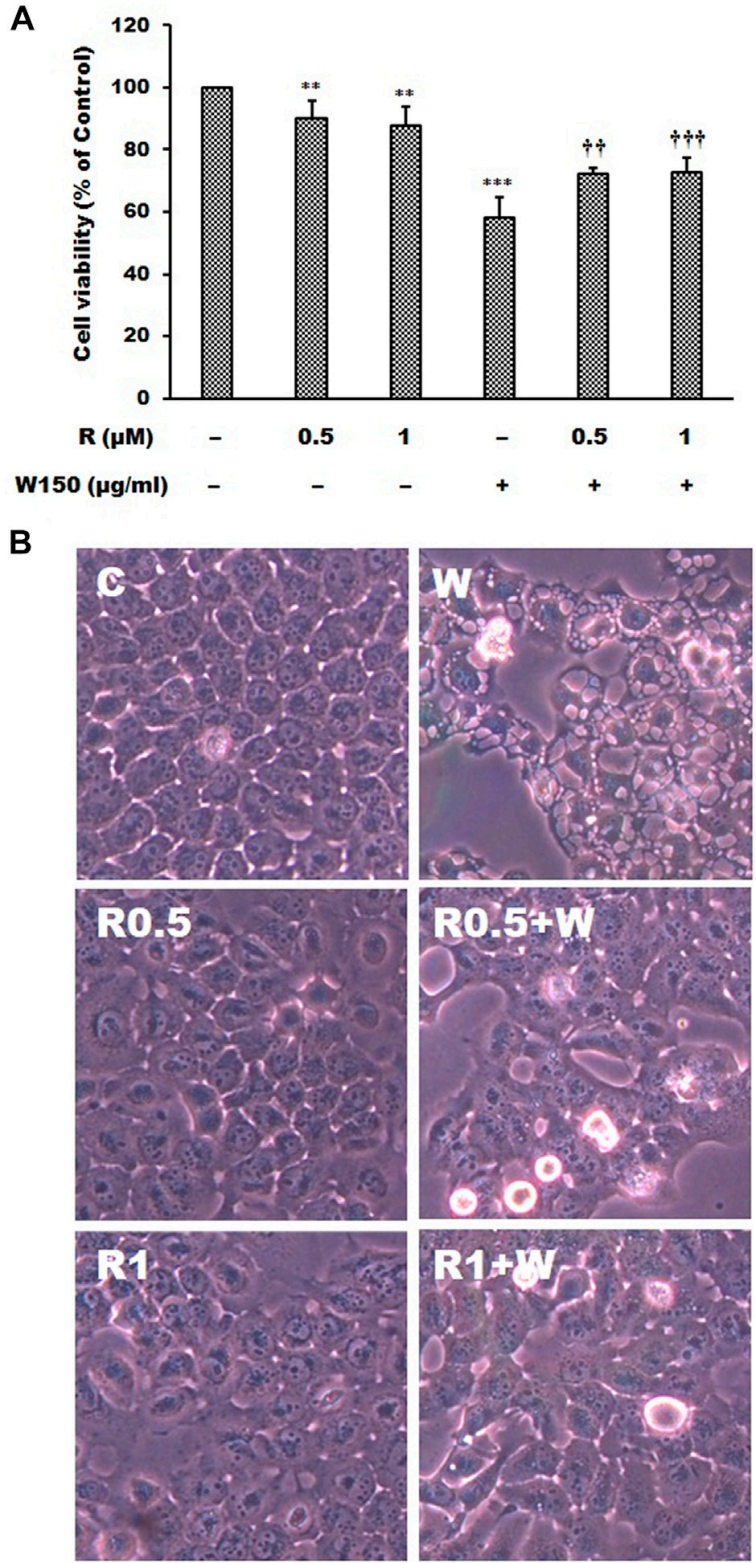
The effects of rapamycin on WGM extracts-induced cell death and membrane-enclosed vacuoles of Hep3B cells. Cells were pretreated with 0.5 or 1 μM rapamycin (R) for 1 h and then treated with 0.1% DMSO or 150 μg/ml WGM extracts (W) for 24 h. **(A)** The effects of rapamycin on WGM extracts-induced cell death of Hep3B cells. After treatment, the viable cells were measured by MTT assay. All determinations are expressed as the mean % control ±S.D. of duplicate from three independent experiments. **p < 0.01, ***p < 0.001 compared to the control values. ^††^ *p* < 0.01, ^†††^ *p* < 0.001 compared to the WGM extracts alone. **(B)** The effects of rapamycin on WGM extracts-induced membrane-enclosed vacuoles of Hep3B cells. After treatment, the cells were immediately photographed with an Olympus IX 73 phase-contrast microscope. All results are representative of three independent experiments.

**FIGURE 6:**
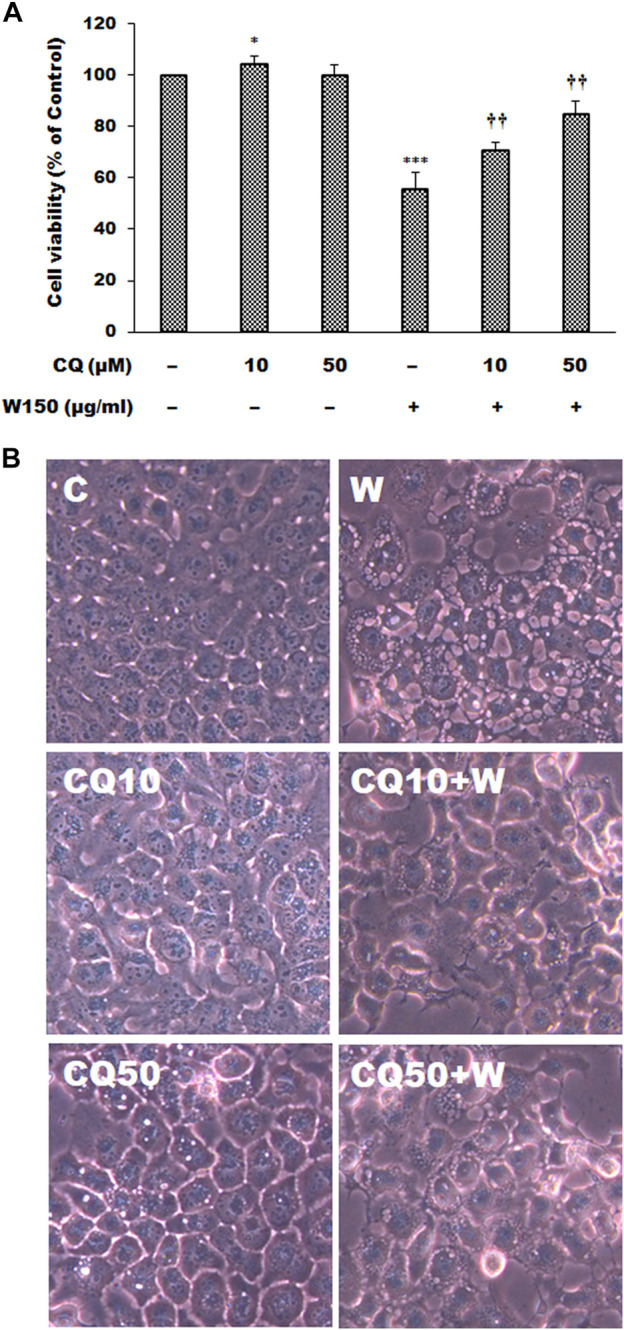
The effects of chloroquine on WGM extracts-induced cell death and membrane-enclosed vacuoles of Hep3B cells. Cells were pretreated with 10 or 50 μM chloroquine (CQ) for 1 h and then treated with 0.1% DMSO or 150 μg/ml WGM extracts (W) for 24 h. **(A)** The effects of chloroquine on WGM extracts-induced cell death of Hep3B cells. After treatment, the viable cells were measured by MTT assay. All determinations are expressed as the mean % control ±S.D. of duplicate from three independent experiments. *p < 0.05, ***p < 0.001 compared to the control values. ^††^ *p* < 0.01 compared to the WGM extracts alone. **(B)** The effects of chloroquine on WGM extracts-induced membrane-enclosed vacuoles of Hep3B cells. After treatment, the cells were immediately photographed with an Olympus IX 73 phase-contrast microscope. All results are representative of three independent experiments.

As shown by the Western blotting analysis, we guess that the cell death of WGM extracts treated Hep3B cells is related to PI3K/Akt/mTOR signaling pathway. Therefore, we used Akt inhibitor MK2206 and mTOR inhibitor rapamycin to demonstrate that PI3K/Akt/mTOR signaling pathway is an important intracellular signaling pathway in the WGM extracts-induced cell death. MK2206, a well-known Akt inhibitor, was used to demonstrate whether Akt activity can promote the WGM extracts-induced cell death of Hep3B cells. The present study demonstrated that MK2206 (0.3 μM) was only slightly restore the cell death induced by WGM extracts (Figure 4A). MK2206 (0.3 and 3 μM, pretreatment 1 h) had a significant preventive effect, however, on the WGM extracts (150 μg/ml)-induced membrane-enclosed vacuoles of Hep3B cells (Figure 4B). The mTOR inhibitor rapamycin, an autophagy inducer, was also used in this study. As shown in Figures 5A,B, a significant preventive effect on the WGM extracts (150 μg/ml)-induced cell death and vacuoles formation was observed in Hep3B cells pretreatment with 0.5 or 1 μM rapamycin. Hep3B cells did not show any cytoplasmic vacuolization after 24 h treatment with 0.5 or 1 μM rapamycin alone, but the proliferation of Hep3B cells were slightly inhibited by the presence of rapamycin (Figure 5A). Our previous study has demonstrated that 3-MA, PI3K inhibitor, did not recover the membrane-enclosed vacuoles and cell death induced by 150 μg/ml WGM extracts (data not shown). These results showed that PI3K/Akt/mTOR signaling pathway was involved in the WGM extracts-induced cell death in Hep3B cells. The lysosomotropic agent chloroquine, an autophagy inhibitor, was also used in this study. The WGM extracts (150 μg/ml)-induced vacuoles formation and cell death of Hep3B cells were partially blocked by pretreatment with 10 or 50 μM chloroquine (Figure 6A). It is interesting to note that chloroquine alone had a slight effect on the vacuoles formation in Hep3B cells (Figure 6B).

### The effects of extracellular signal-regulated kinase inhibitor LY3214996 and p38 inhibitor SB202190 on white genius mushroom extracts-induced cell death and membrane-enclosed vacuoles of Hep3B cells

**FIGURE 7:**
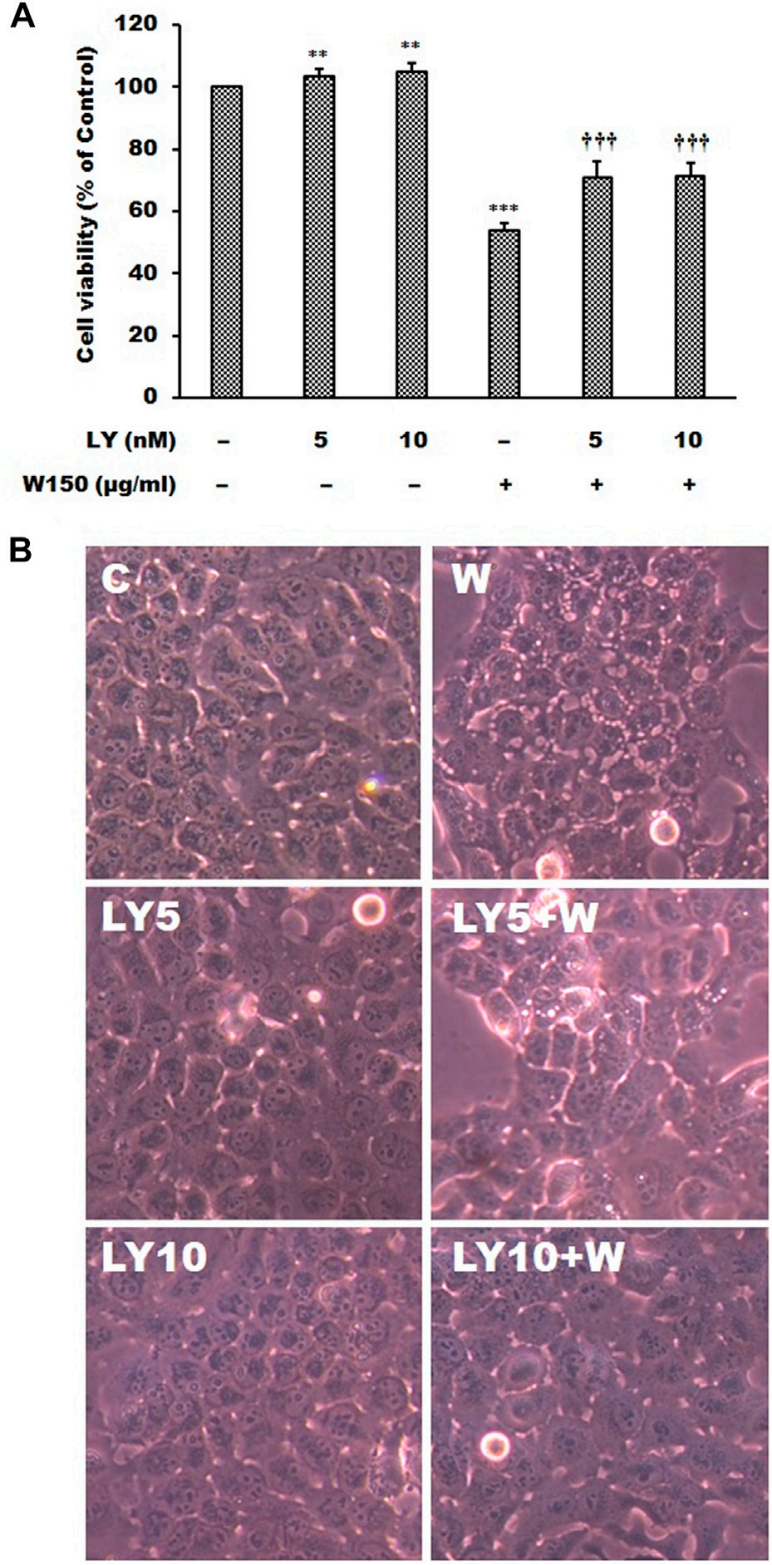
The effects of LY3214996 on WGM extracts-induced cell death and membrane-enclosed vacuoles of Hep3B cells. Cells were pretreated with 5 or 10 nM LY3214996 (LY) for 1 h and then treated with 0.1% DMSO or 150 μg/ml WGM extracts (W) for 24 h. **(A)** The effects of LY3214996 on WGM extracts-induced cell death of Hep3B cells. After treatment, the viable cells were measured by MTT assay. All determinations are expressed as the mean % control ±S.D. of duplicate from three independent experiments. **p < 0.01, ***p < 0.001 compared to the control values. ^†††^ *p* < 0.001 compared to the WGM extracts alone. **(B)** The effects of LY3214996 on WGM extracts-induced membrane-enclosed vacuoles of Hep3B cells. After treatment, the cells were immediately photographed with an Olympus IX 73 phase-contrast microscope. All results are representative of three independent experiments.

**FIGURE 8:**
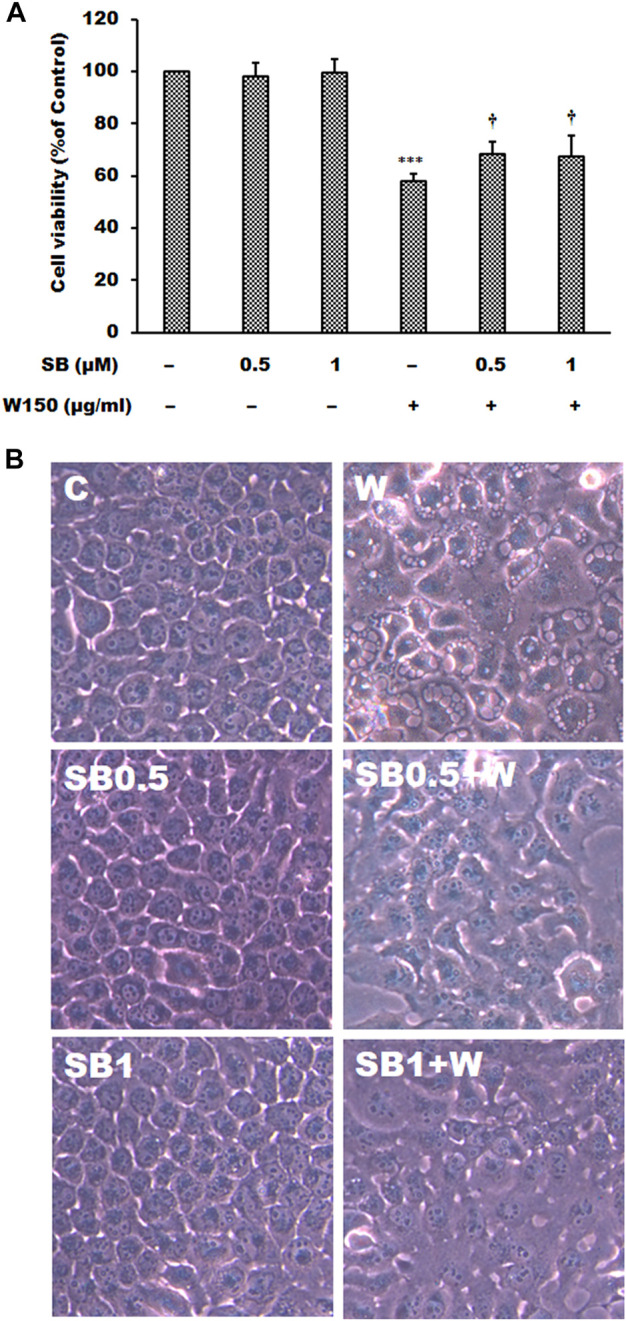
The effects of SB202190 on WGM extracts-induced cell death and membrane-enclosed vacuoles of Hep3B cells. Cells were pretreated with 0.5 or 1 μM SB202190 (SB) for 1 h and then treated with 0.1% DMSO or 150 μg/ml WGM extracts (W) for 24 h. **(A)** The effects of SB202190 on WGM extracts-induced cell death of Hep3B cells. After treatment, the viable cells were measured by MTT assay. All determinations are expressed as the mean % control ±S.D. of duplicate from three independent experiments. ***p < 0.001 compared to the control values. ^†^ *p* < 0.05 compared to the WGM extracts alone. **(B)** The effects of SB202190 on WGM extracts-induced membrane-enclosed vacuoles of Hep3B cells. After treatment, the cells were immediately photographed with an Olympus IX 73 phase-contrast microscope. All results are representative of three independent experiments.

It is well known that MAPK signaling pathway was an important intracellular signaling pathway that is related to cell proliferation, motility, survival and apoptosis of cancer cells. According to the results of immunoblotting, WGM extracts caused a marked increase in the protein levels of pERK in Hep3B cells. The ERK inhibitor LY3214996 was used in this study. Pretreatment with LY3214996 (5 and 10 nM) significantly inhibited the WGM extracts (150 μg/ml)-induced cell death and membrane-enclosed vacuoles of Hep3B cells (Figure 7). In this study, not only the expression of phosphorylation of ERK but also phosphorylation of p38 is regulated by WGM extracts according to the results of immunoblotting. This study determined the effect of p38 inhibitor SB202190 on the WGM extracts-induced cell death of Hep3B cells. Pretreatment with SB202190 (0.5 and 1 μM) for 1 h had a significant preventive effect on the WGM extracts (150 μg/ml)-induced cell death and membrane-enclosed vacuoles of Hep3B cells (Figure 8). Based on the above data, these results indicate that WGM extracts-mediated the activation of ERK and p38 is involved in the WGM extracts-induced membrane-enclosed vacuoles and even cell death.

### The effect of white genius mushroom extracts on the migration potential of Hep3B cells

**FIGURE 9:**
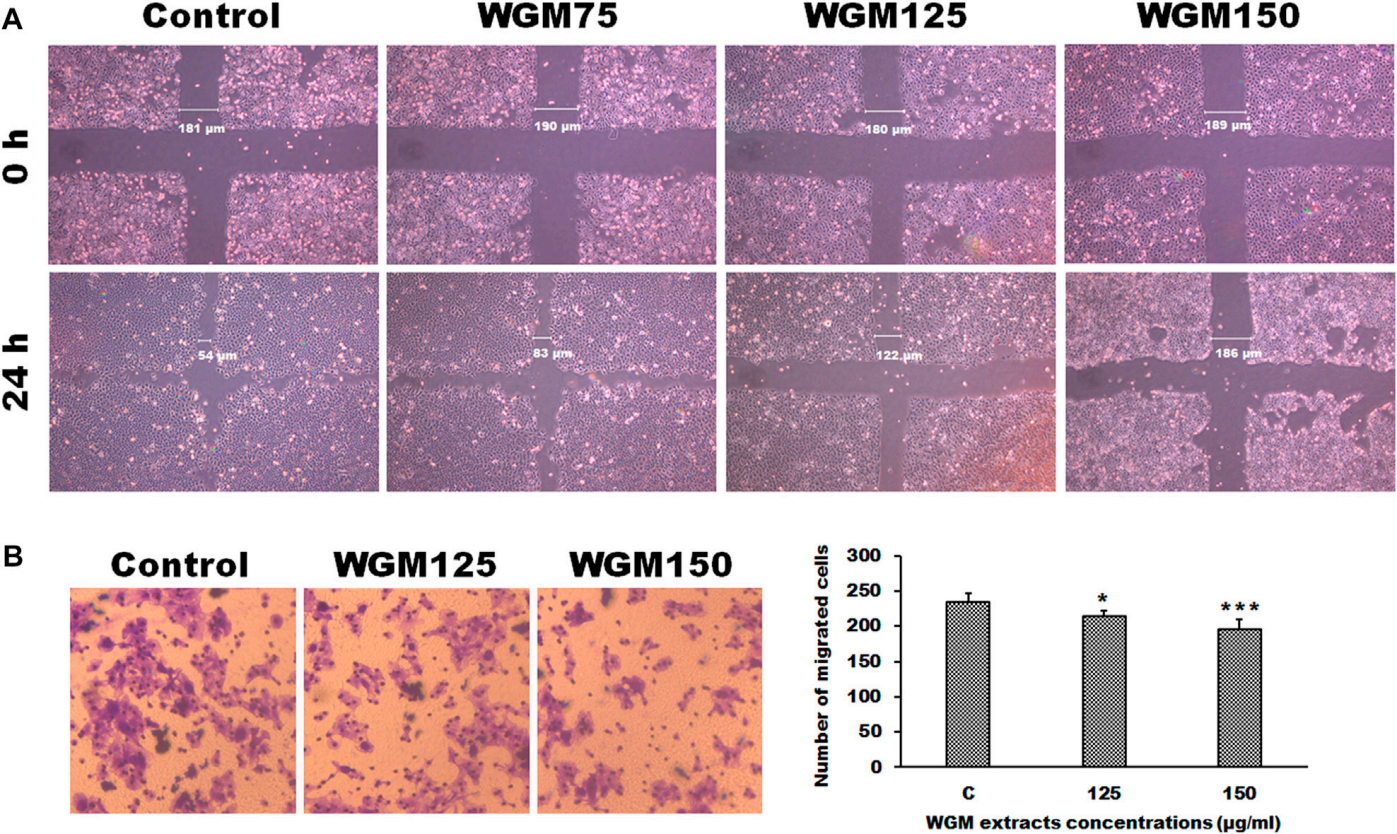
The effect of WGM extracts on cell migration of Hep3B cells. **(A)** The migratory activity of Hep3B cells was assessed using a wounded migration assay. A linear wound was made with a sterile 200 μl pipette tip. Cells were treated with vehicle alone (Control) or with 75, 125 or 150 μg/ml WGM extracts for 24 h. Images of wounds at 0 h and 24 h after scratching were obtained with an Olympus IX 73 phase-contrast microscope at objective ×10 magnification. All results are representative of three independent experiments. **(B)** The effects of WGM extracts on cell migration of Hep3B cells were also measured by Transwell assay in the study. Cell migration was also detected using Transwell assay with a pore size of 8 μm the Transwell inserts for 24-well plates. Cells were treated with vehicle alone or with 125 or 150 μg/ml WGM extracts for 24 h. Filters were fixed with 4% paraformaldehyde solution for 20 min and stained with 0.1% crystal violet for 10 min, then the cells that passed through the filter were photographed by Olympus IX 73 phase-contrast microscope at objective ×20 magnification. Left: Representative images of Transwell migration assay. Right: Cells were counted from 4 random microscope fields for each sample in three independent experiments. Results are expressed as the mean ± S.D. *p < 0.05, ***p < 0.001 compared to the control values.

Controlling the invasion and metastasis of cancer cells has been recognized as a new strategy for cancer prevention and treatment. The MAPK and mTOR signaling pathway are most important signaling cascade and play key role in the proliferation and migration of tumor cells. Since cell migration is an essential step in the cancer metastatic process, the present study investigated the effect of WGM extracts on the migration ability of Hep3B cells. For examination of the ability of Hep3B cell migration, a wound-healing assay was performed to examine whether WGM extracts can inhibit Hep3B cell migration. Wound healing experiment was performed on cells treated with 75, 125 and 150 μg/ml WGM extracts for 24 h. As shown in Figure 9A, an increase in the distance of the wound edge indicates that the speed of cell migration is significantly reduced after treatment with WGM extracts. To obtain further support for the inhibition of cell migration by WGM extracts in Hep3B cells, the transwell migration assay, which is used to examine the migratory response of cancer cells to treatments, were performed in this study. The results from that assay showed that WGM extracts had a significant inhibition of the Hep3B cell migration ability in a dose-dependent manner through the transwell membrane compared with those in the control cells (Figure 9B). Based on the above data, we suggested that WGM extracts be able to inhibit Hep3B cell migration in a dose-dependent manner.

### White genius mushroom extracts inhibits the proliferation of Hep3B cells *in vitro*

**FIGURE 10:**
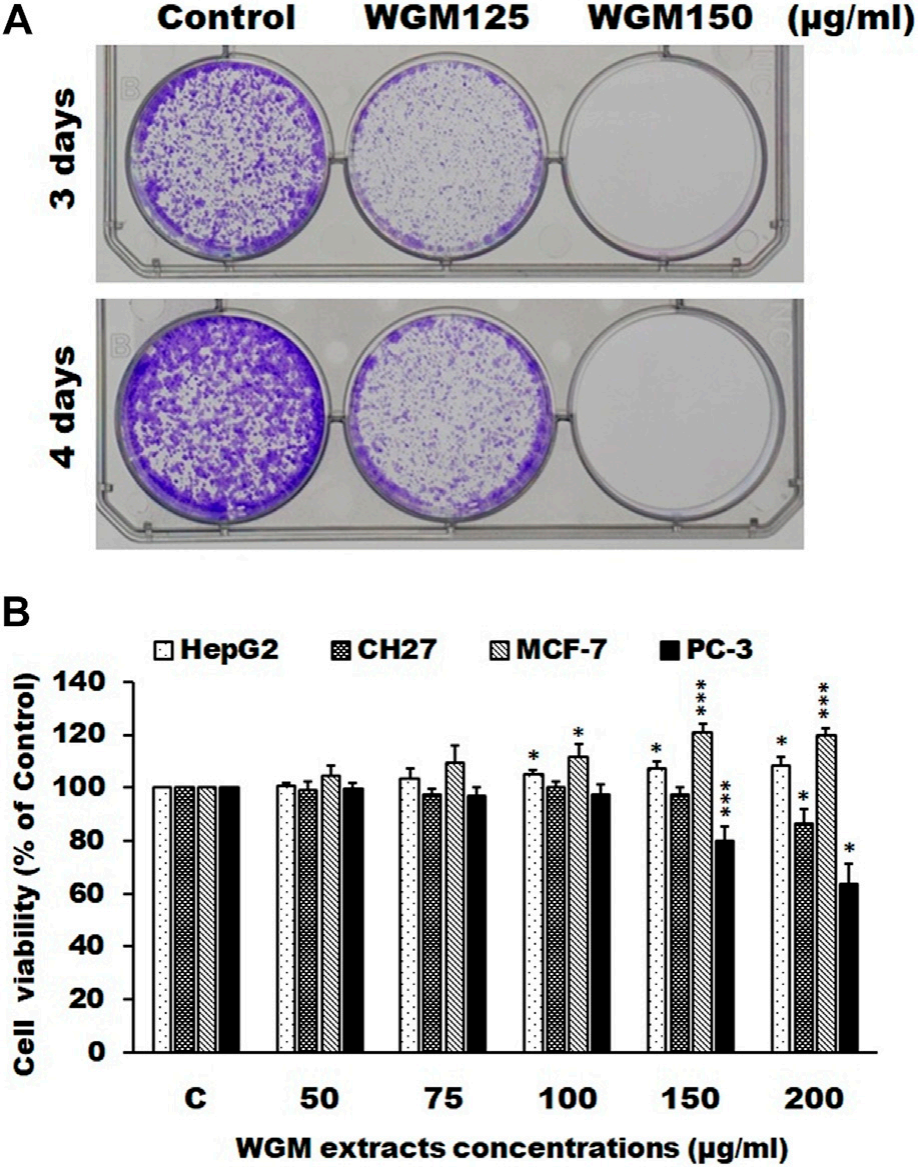
**(A)** WGM extracts inhibits colony forming ability of Hep3B cells in a dose- and time-dependent manner. Representative images of Hep3B colonies after treatment with 125 or 150 μg/ml of WGM extracts. Colonies were fixed with 4% paraformaldehyde solution and then stained with 0.5% crystal violet. Finally, the colonies were photographed. All results are representative of three independent experiments. **(B)** Cytotoxic effects of WGM extracts on HepG2, CH27, MCF-7 and PC-3 cells. HepG2, CH27, MCF-7 and PC-3 cells were incubated with vehicle alone or with various concentrations of WGM extracts for 24 h. After treatment, the viable cells were measured by MTT assay. The data are expressed as the mean % control ±S.D. of three independent experiments performed in duplicate. *p < 0.05, ***p < 0.001 compared to the control values.

Colony formation assay was used to mimic cancer growth from a single cell to grow into a cancer cell colonies or tumor mass. Plate colony formation assay was used to examine the effect of WGM extracts on the colony-forming ability of Hep3B cells. Cells were plated onto 6-well plates and incubated with vehicle alone or with 125 or 150 μg/ml WGM extracts and treated cultures were maintained in culture for an additional 3 or 4 days to allow formation of colonies. As shown in Figure 10A, WGM extracts significantly inhibited the colony-forming ability of Hep3B cells in a dose- and time-dependent manner, as fewer colonies were observed in cells treated with higher concentrations of WGM extracts. In other words, higher doses inhibited colony formation and resulted in a significant decrease in colony numbers in the WGM extracts-treated group. In control group, Hep3B cells grow and form colonies within 3 or 4 days of incubation (Figure 10A). In our previous study, we have confirmed the cytotoxicity of white genius mushroom extracts on human Hep3B liver cancer cells. WGM extracts induced a significant concentration-dependent inhibition of Hep3B cell growth with IC_50_ (half maximal inhibitory concentration) value of 175 μg/ml. We further investigated whether WGM extracts selectively causes cytotoxicity in different cancer cell lines. Therefore, this study evaluated the effects of WGM extracts on cell growth of hepatocellular carcinoma HepG2 cells, human lung squamous carcinoma CH27 cells, breast cancer MCF-7 cells and prostatic adenocarcinoma PC-3 cells. WGM extracts had no significant cytotoxic effect on the HepG2, CH27 and MCF-7 cells after treatment with WGM extracts for 24 h (Figure 10B). Furthermore, WGM extracts treatment leads to an increase in cell viability of MCF-7 cells (Figure 10B). The treatment of PC-3 cells with WGM extracts for 24 h resulted in a slight cytotoxic effect (Figure 10B). The concentration of inducing about 50% cell death by WGM extracts is more than 200 μg/ml for PC-3 cells (Figure 10B). Based on the above data, we demonstrated that the cytotoxic effect of WGM extracts on Hep3B cells was significantly higher than that in PC-3, HepG2, CH27 and MCF-7 cells. Therefore, Hep3B cells were chosen for investigation of the anticancer mechanisms of WGM extracts in this study.

## Discussion

Hepatocellular carcinoma (HCC) is one of the most common cancers in Taiwan, with a high incidence and mortality rate. However, the drugs used to treat for liver cancer may appear high toxicity, many side effects and drug resistance, leading to poor quality of life after treated for cancer. Therefore, the search for new drugs or chemoprevention agents with few side effects and high efficacy is in demand in order to treat liver cancer patients. White genius mushroom (WGM) is a popular edible mushroom whose anticancer activity was demonstrated to be partially dependent on the production of ROS in human Hep3B liver cancer cells in our previous study (Li et al., 2022). Furthermore, autophagy, mitophagy and apoptosis pathways were identified as significant in WGM extracts-treated Hep3B liver cancer cells according to the KEGG pathway enrichment analysis (Li et al., 2022). Although WGM extracts were found to have anticancer activities, the exact mechanisms of the anticancer effect of WGM extracts are not fully understood. In this study, WGM extracts was evaluated for its anticancer activities and exact mechanisms in hepatocellular carcinoma Hep3B cells. Since WGM is a pharmacologically safe natural agent, WGM might be a dietary chemopreventive agent for cancer treatment with an excellent safety profile *in vivo*.

RNA expression profiles produced by NGS technology allow comprehensive investigation of transcribed sequences in distinct cellular states (Wu et al., 2019; Shi et al., 2020). The KEGG pathways database is the comprehensive coverage of a wide range of different biochemical pathways. In general, KEGG pathway enrichment analysis was conducted to identify differential expressed genes (DEGs) into significantly enriched metabolic pathways or signaling pathways. In this study, the KEGG pathway analysis was used to screen the enrichment of dysfunctional signaling pathways of the DEGs in WGM extracts-treated cells. KEGG pathway enrichment analysis showed that the DEGs induced by WGM were found to be involved in organismal systems, metabolism, human diseases, genetic information processing, environmental information processing and cellular process. In the environmental information processing of KEGG pathway analysis, Hippo, mTOR, FoxO, MAPK and TGF-beta signaling pathways were significantly changed. The Hippo signaling pathway is a highly conserved tumor suppressor pathway that exerts a critical role in modulating cell division, cell proliferation and apoptosis. It has been suggested that Hippo signaling pathway is a potent growth and tumor suppressor pathway in the mammalian liver (Lu et al., 2010; Hong et al., 2015). FoxOs (forkhead box class O proteins) are also considered to be tumor suppressors and known to be implicated in the progression of several human cancers (Su et al., 2014; Jiramongkol and Lam, 2020). The mTOR and MAPK signaling pathways are necessary to promote growth and proliferation in many cancer cell types. PI3K/Akt/mTOR and MAPK signaling pathways had been demonstrated to be involve in cell proliferation, motility, survival and apoptosis of cancer cells (Chen, 2010; Ma et al., 2013; Zhang et al., 2016). Recently, PI3K/Akt/mTOR and Ras/Raf/MEK/ERK signaling pathways have also been recognized as a new strategy for cancer therapy (Asati et al., 2016). Therefore, we focused our attention on the investigation of the effects of WGM extracts on the expression of mTOR and MAPK signaling pathways in Hep3B cells in this study.

This study demonstrated that PI3K/Akt/mTOR signaling pathway was involved in the WGM extracts-induced cell death in Hep3B cells according to the results of the Western blotting analysis. Wu et al. (2010) demonstrated that PI3K inhibitor 3-MA can block autophagosome formation by inhibiting PI3K, thereby inhibiting autophagy. However, 3-MA did not recover the membrane-enclosed vacuoles and cell death induced by WGM extracts in our previous study (Li et al., 2022). In this study, we demonstrated that Akt inhibitor MK2206 was only slightly restore the cell death induced by WGM extracts. MK2206 had a significant preventive effect, however, on the WGM extracts-induced membrane-enclosed vacuoles of Hep3B cells. Furthermore, pretreatment with the mTOR inhibitor rapamycin abolished the WGM extracts-induced cell death and membrane-enclosed vacuoles of Hep3B cells in this study. It is interesting to note that MK2206 did not have as much effect on the WGM extracts-induced cell death as rapamycin did. In addition to 3-MA, we demonstrated that the inhibitors of PI3K/Akt/mTOR signaling pathway are able to prevent the WGM-induced vacuoles formation and cell death, indicating PI3K/Akt/mTOR signaling pathway is an important intracellular signaling pathway in the WGM extracts-induced cell death. In this study, the Akt and mTOR inhibitor can reverse the WGM extracts-mediated Hep3B cell death of Hep3B cells which are consistent with those of other studies reporting that the dependence of PI3K/Akt/mTOR signaling on the growth and survival of certain tumors has broad implications in cancer therapy (Guertin and Sabatini, 2007; Fasolo and Sessa, 2008; Zhou et al., 2011; Hassan et al., 2013). It is worthy of note that mTOR inhibitor is significantly more effective in preventing the membrane-enclosed vacuoles formation and cell death induced by WGM extracts than PI3K or Akt inhibitors.

In this study, we demonstrated that mTOR inhibitor rapamycin actually reversed WGM-induced cell death and vacuole formation. However, rapamycin is also a well-known autophagy inducer, which activates autophagy by repressing the mechanistic target of mTOR which is associated with lysosomal biogenesis and autophagosome-lysosome fusion (Huang et al., 2021). This study also investigated whether chloroquine could inhibit the induction of cell death and vacuole formation induced by WGM of Hep3B cells. Chloroquine, which is lysosomotropic agent, is a classic inhibitor of autophagy that blocks autophagic processes by inhibiting the binding of autophagosomes to lysosomes (Mushtaque and Shahjahan, 2015). Furthermore, lysosomotropic agents are one of the triggers for cytoplasmic vacuolation caused by lysosomal dysfunction (Zou et al., 2020). The present study showed that chloroquine had a significant preventive effect on the WGM extracts-induced cell death of Hep3B cells. It is interesting to note that both the autophagy inducer rapamycin and the autophagy inhibitor chloroquine prevented WGM-induced membrane-enclosed vacuoles and cell death in Hep3B cells. We also demonstrated that chloroquine was more effective than rapamycin in preventing WGM extract-induced cell death, however the prevention of vacuole formation had the opposite effect. According to the results of immunoblotting, WGM extracts induced a marked increase in the expression of the mTOR protein. It indicated that WGM might attempt to inhibit autophagy process by activating mTOR protein expression. Therefore, we guessed that rapamycin may inhibit WGM-induced cell death through induction autophagy, which is essential for maintaining cell survival. While autophagy is considered as cell survival mechanism, the occurrence of autophagy is also thought to promote apoptosis and even accelerate cell death (Gump and Thorburn, 2011; Rubinstein et al., 2011; Mukhopadhyay et al., 2014; Wei et al., 2014). Since chloroquine prevents WGM extract-induced cell death more efficiently than rapamycin, we further confirmed that Hep3B cells should attempt to survive by inducing autophagy during WGM extracts-induced death cells in this study.

The most important pathway interacting with PI3K/Akt in different types of cancers is the Ras/Raf/ERK pathway (Castellano and Downward, 2011). MAPK signaling pathway signaling pathway was found to be a crucial intracellular signaling pathway, which is closely related to proliferation, apoptosis and metastasis of cancer cells (You et al., 2011; Guo et al., 2016). According to the results of immunoblotting, WGM extracts induced a marked dose-dependent decrease in the expression of the ERK protein, and an upregulation of the Ras, Raf and MEK protein in this study. The expression patterns of pERK protein are similar to those seen in Akt and pAkt expression after treatment with WGM extracts for 24 h in this study. There is abundant evidence that PI3K/Akt has surprisingly extensive cross talk with ERK1/2 (Yang et al., 2019; Duff et al., 2021). Furthermore, PI3K/Akt and ERK1/2 pathways are downstream of EGF-EGFR signaling in many cell types (Zhang et al., 2012; Fan et al., 2015; Li et al., 2019). We also demonstrated that treatment with WGM extracts increased pERK protein in Hep3B cells up to 125 μg/ml, but pERK level was decreased after treatment with 175 μg/ml WGM extracts. It is interesting to note that WGM extracts induced a significant increase in the protein expression of Ras in cells exposed to 175 μg/ml WGM extracts for 24 h. These results indicate that WGM extracts-mediated ERK activation occurs through a Ras-independent pathway. It had also been reported that the crosstalk between ERK and Akt is mediated by EGFR and independent of Ras or Raf mutation (Klinger et al., 2013). In addition to the ERK, p38 is also an important intracellular signaling pathway in the MAPK signaling pathway. In our study, not only the expression of ERK, which is associated with cell proliferation, but also p38, which is regulated by various environmental stresses, is regulated by WGM extracts. As shown by immunoblotting, WGM extracts caused a marked increase in the protein levels of pp38, but p38 levels decrease in Hep3B cells. According to the results of immunoblotting, the ERK inhibitor LY3214996 and p38 inhibitor SB202190 were used in this study. The present study showed that both inhibitors partially abolished the WGM extracts-induced cell death and membrane-enclosed vacuoles of Hep3B cells. It indicate that WGM extracts-mediated the activation of ERK and p38 is involved in the WGM extracts-induced membrane-enclosed vacuoles and even cell death. Based on the above data, we also demonstrated that MAPK pathway is an important intracellular signaling pathway in the WGM extracts-induced cell death.

Our previous study indicated that only (2*E*,6*E*)-3,7,11,15,19,23,27,31,35-non-amethylhexatriaconta-2,6,34-triene-1,11,15,19,23,27,31-heptol and (18:2) lysophosphatidylcholine (lysoPC) were identified in the WGM alcoholic extracts using UPLC-MS/MS analyses, MS data processing and Molecular networking–GNPS (http://gnps.ucsd.edu) (Li et al., 2022). (2*E*,6*E*)-3,7,11,15,19,23,27,31,35-non-amethylhexatriaconta-2,6,34-triene-1,11,15,19,23,27,31-heptol has been considered to be an inhibitor of the NK-1 (neurokinin-1) and NK-2 (neurokinin-2) receptors (Hegde et al., 1997). Recently, the NK-1 receptor (NK1R) was demonstrated to be involved in the development of the various cancer cells (Muñoz and Coveñas, 2014; Ebrahimi et al., 2020; Isorna et al., 2020). It has also been suggested that NK1R is overexpressed in human liver cancer and can significantly inhibit the proliferation of cancer cells by antagonizing the expression of NK1R (Garnier et al., 2016; Muñoz et al., 2019). All these reports suggest that NK-1R plays an important role in regulation cancer cell proliferation, apoptosis and migration for invasion or metastasis. Furthermore, PI3K/Akt/mTOR and MAPK signaling pathways was demonstrated to have a crucial role in the cell growth inhibition and apoptosis exerted by NK-1R antagonists (Castagliuolo et al., 2000; Garnier et al., 2016; Shi et al., 2021). In general, the cancer cell proliferation, motility, survival and apoptosis associated with PI3K/Akt/mTOR and MAPK signaling pathways have been well documented. Therefore, we guessed that WGM extracts might regulate PI3K/Akt/mTOR and MAPK signaling pathways and induce apoptosis and migration through blocking NK-1R in Hep3B cells.

Migration and invasion are important factors that accelerate the occurrence and progression of malignant tumors. Many cellular signaling pathways are thought to be involved in the proliferation and migration of cancer cells (Guo et al., 2016; Luo et al., 2016; Yang et al., 2016). PI3K/Akt and MAPK signaling pathways have been reported to be important signaling pathways involved in the network regulation on cell migration in many different kinds of cancer (Guo et al., 2016; Luo et al., 2016; Yang et al., 2016). According to the results of immunoblotting and experiments of inhibitors, this study examined whether WGM extracts will yield an inhibitory effect on cell migration of Hep3B cells. To obtain further support for the inhibition of cell migration by WGM extracts in Hep3B cells, the transwell migration and wound healing assays, which are used to examine the migratory response of cancer cells to treatments, were performed in this study. The results from those assays showed that WGM extracts had a significant inhibition of the Hep3B cell migration ability in a dose-dependent manner. Colony formation assay was used to mimic cancer growth from a single cell to grow into a cancer cell colonies or tumor mass. Plate colony formation assay was used to examine the effect of WGM extracts on the colony-forming ability of Hep3B cells. We demonstrated that WGM extracts had a significant inhibitory effect on the colony forming ability of Hep3B cells in a concentration- and time-dependent manner. Based on the above data, we suggest that PI3K/Akt/mTOR and MAPK signaling pathways be involved in the WGM extracts-mediated inhibition of cell colony formation and migration of Hep3B cells. It is worthy to note that WGM extracts selectively induced cytotoxicity in different cancer cell lines. We demonstrated that the cytotoxic effect of WGM extracts on Hep3B cells was significantly higher than that in prostate cancer PC-3 cells, liver cancer HepG2, lung cancer CH27 and breast cancer MCF-7 cells.

## Conclusion

According to the KEGG pathway enrichment analysis, the results of immunoblotting and experiments of inhibitors, we demonstrated that PI3K/Akt/mTOR and MAPK signaling pathways were involved in WGM extracts-induced vacuoles formation and cell death. Our results are the first findings to indicate that WGM extracts induces cell death in Hep3B cancer cells and that the induction of vacuoles formation and cell death coincides with the PI3K/Akt/mTOR and MAPK signaling pathways. This study also demonstrated that WGM extracts had a significant inhibition of the Hep3B cell colony formation and migration ability in a dose-dependent manner. The present findings indicate that WGM should be a pharmacologically safe natural agent and might be a dietary chemopreventive agent for the cancer treatment.

## Data Availability

The raw data supporting the conclusions of this article will be made available by the authors, without undue reservation. The RNA sequencing raw data was uploaded to NCBI Sequence Read Archive (SRA), and the accession ID is PRJNA813700.
