# Supplementary material for: Transcriptome analysis revealed the role of mTOR and MAPK signaling pathways in the white strain of Hypsizygus marmoreus extracts-induced cell death of human hepatoma Hep3B cells
Source: Front Pharmacol. 2022 Nov 25;13:1039376. doi: 10.3389/fphar.2022.1039376 (PMC9732266; doi:10.3389/fphar.2022.1039376)
Supplement: Supplementary file 1 [file Table1.DOCX]

Supplementary Data

| **Table S1 The differentially expressed genes involved in mTOR signaling pathway** | | | |
| --- | --- | --- | --- |
| GeneID | GeneSymbol | Regulation | KO |
| \| ENSG00000100485 \| \| --- \| \| ENSG00000115596 \| \| ENSG00000116039 \| \| ENSG00000104290 \| \| ENSG00000135925 \| \| ENSG00000117461 \| \| ENSG00000143816 \| \| ENSG00000154764 \| \| ENSG00000111186 \| \| ENSG00000176641 \| \| ENSG00000180340 \| \| ENSG00000104365 \| \| ENSG00000177283 \| \| ENSG00000168003 \| \| ENSG00000204673 \| \| ENSG00000155097 \| \| ENSG00000115904 \| \| ENSG00000135930 \| \| ENSG00000105221 \| \| ENSG00000140992 \| \| ENSG00000051382 \| \| ENSG00000174804 \| \| ENSG00000177169 \| \| ENSG00000100485 \| \| ENSG00000266173 \| \| \| ENSG00000106615 \| \| --- \| \| ENSG00000157764 \| \| ENSG00000082701 \| \| ENSG00000187840 \| \| ENSG00000047249 \| \| ENSG00000067182 \| \| ENSG00000213341 \| \| ENSG00000116954 \| \| ENSG00000107404 \| \| ENSG00000107404 \| \| ENSG00000137154 \| \| ENSG00000137154 \| \| ENSG00000167965 \| \| ENSG00000154803 \| \| ENSG00000154803 \| \| ENSG00000177885 \| \| ENSG00000108443 \| \| ENSG00000115904 \| \| ENSG00000174775 \| \| ENSG00000142208 \| \| ENSG00000165699 \| \| ENSG00000135932 \| \| ENSG00000132155 \| \| ENSG00000063046 \| \| ENSG00000264187 \| \| ENSG00000132155 \| \| ENSG00000180747 \| \| ENSG00000103197 \| \| ENSG00000103197 \| \| ENSG00000134248 \| \| ENSG00000154803 \| \| ENSG00000100068 \| \| ENSG00000157764 \| \| ENSG00000107404 \| \| ENSG00000106615 \| \| ENSG00000171862 \| \| ENSG00000108443 \| \| ENSG00000118515 \| \| ENSG00000108443 \| \| ENSG00000161202 \| \| ENSG00000154803 \| \| ENSG00000106615 \| \| ENSG00000105221 \| \| ENSG00000108443 \| \| ENSG00000165699 \| \| ENSG00000132155 \| \| ENSG00000137154 \| \| ENSG00000151247 \| \| ENSG00000104365 \| \| ENSG00000155097 \| \| ENSG00000147416 \| \| ENSG00000175634 \| \| ENSG00000175634 \| \| ENSG00000175634 \| \| ENSG00000162337 \| \| ENSG00000175634 \| \| ENSG00000168003 \| \| ENSG00000168003 \| \| ENSG00000177169 \| \| ENSG00000168003 \| \| ENSG00000105221 \| \| ENSG00000168003 \| \| ENSG00000168003 \| \| ENSG00000168003 \| \| ENSG00000168003 \| \| ENSG00000168003 \| \| ENSG00000168003 \| \| ENSG00000177189 \| \| ENSG00000168003 \| \| ENSG00000063046 \| \| ENSG00000063046 \| \| ENSG00000063046 \| \| ENSG00000258186 \| \| ENSG00000142208 \| \| ENSG00000142208 \| \| ENSG00000133703 \| \| ENSG00000142208 \| \| ENSG00000167965 \| \| ENSG00000167965 \| \| ENSG00000167965 \| \| ENSG00000141564 \| \| ENSG00000004975 \| \| ENSG00000004975 \| \| ENSG00000154229 \| \| ENSG00000108443 \| \| ENSG00000108443 \| \| ENSG00000213341 \| \| | \| SOS2 \| \| --- \| \| WNT6 \| \| ATP6V1B1 \| \| FZD3 \| \| WNT10A \| \| PIK3R3 \| \| WNT9A \| \| WNT7A \| \| WNT5B \| \| RNF152 \| \| FZD2 \| \| IKBKB \| \| FZD8 \| \| SLC3A2 \| \| AKT1S1 \| \| ATP6V1C1 \| \| SOS1 \| \| EIF4E2 \| \| AKT2 \| \| PDPK1 \| \| PIK3CB \| \| FZD4 \| \| ULK1 \| \| SOS2 \| \| STRADA \| \| \| RHEB \| \| --- \| \| BRAF \| \| GSK3B \| \| EIF4EBP1 \| \| ATP6V1H \| \| TNFRSF1A \| \| CHUK \| \| RRAGC \| \| DVL1 \| \| DVL1 \| \| RPS6 \| \| RPS6 \| \| MLST8 \| \| FLCN \| \| FLCN \| \| GRB2 \| \| RPS6KB1 \| \| SOS1 \| \| HRAS \| \| AKT1 \| \| TSC1 \| \| CAB39 \| \| RAF1 \| \| EIF4B \| \| RP11-45M22.4 \| \| RAF1 \| \| CTD-2547E10.2 \| \| TSC2 \| \| TSC2 \| \| LAMTOR5 \| \| FLCN \| \| LRP5L \| \| BRAF \| \| DVL1 \| \| RHEB \| \| PTEN \| \| RPS6KB1 \| \| SGK1 \| \| RPS6KB1 \| \| DVL3 \| \| FLCN \| \| RHEB \| \| AKT2 \| \| RPS6KB1 \| \| TSC1 \| \| RAF1 \| \| RPS6 \| \| EIF4E \| \| IKBKB \| \| ATP6V1C1 \| \| ATP6V1B2 \| \| RPS6KB2 \| \| RPS6KB2 \| \| RPS6KB2 \| \| LRP5 \| \| RPS6KB2 \| \| SLC3A2 \| \| SLC3A2 \| \| ULK1 \| \| SLC3A2 \| \| AKT2 \| \| SLC3A2 \| \| SLC3A2 \| \| SLC3A2 \| \| SLC3A2 \| \| SLC3A2 \| \| SLC3A2 \| \| RPS6KA3 \| \| SLC3A2 \| \| EIF4B \| \| EIF4B \| \| EIF4B \| \| SLC7A5P2 \| \| AKT1 \| \| AKT1 \| \| KRAS \| \| AKT1 \| \| MLST8 \| \| MLST8 \| \| MLST8 \| \| RPTOR \| \| DVL2 \| \| DVL2 \| \| PRKCA \| \| RPS6KB1 \| \| RPS6KB1 \| \| CHUK \| \| | \| Down \| \| --- \| \| Down \| \| Down \| \| Down \| \| Down \| \| Down \| \| Down \| \| Down \| \| Down \| \| Down \| \| Down \| \| Down \| \| Down \| \| Down \| \| Down \| \| Down \| \| Down \| \| Down \| \| Down \| \| Down \| \| Down \| \| Down \| \| Down \| \| Down \| \| Down \| \| \| Ups \| \| --- \| \| Ups \| \| Ups \| \| Ups \| \| Ups \| \| Ups \| \| Ups \| \| Ups \| \| Ups \| \| Ups \| \| Ups \| \| Ups \| \| Ups \| \| Ups \| \| Ups \| \| Ups \| \| Ups \| \| Ups \| \| Ups \| \| Ups \| \| Ups \| \| Ups \| \| Ups \| \| Ups \| \| Ups \| \| Ups \| \| Ups \| \| Ups \| \| Ups \| \| Ups \| \| Ups \| \| Ups \| \| Ups \| \| Ups \| \| Ups \| \| Ups \| \| Ups \| \| Ups \| \| Ups \| \| Ups \| \| Ups \| \| Ups \| \| Ups \| \| Ups \| \| Ups \| \| Ups \| \| Ups \| \| Ups \| \| Ups \| \| Ups \| \| Ups \| \| Ups \| \| Ups \| \| Ups \| \| Ups \| \| Ups \| \| Ups \| \| Ups \| \| Ups \| \| Ups \| \| Ups \| \| Ups \| \| Ups \| \| Ups \| \| Ups \| \| Ups \| \| Ups \| \| Ups \| \| Ups \| \| Ups \| \| Ups \| \| Ups \| \| Ups \| \| Ups \| \| Ups \| \| Ups \| \| Ups \| \| Ups \| \| Ups \| \| Ups \| \| Ups \| \| Ups \| \| Ups \| \| Ups \| \| Ups \| \| Ups \| \| Ups \| \| | \| K03099 \| \| --- \| \| K00445 \| \| K02147 \| \| K02329 \| \| K01357 \| \| K02649 \| \| K01064 \| \| K00572 \| \| K00444 \| \| K15705 \| \| K02235 \| \| K07209 \| \| K02375 \| \| K06519 \| \| K16184 \| \| K02148 \| \| K03099 \| \| K03259 \| \| K04456 \| \| K06276 \| \| K00922 \| \| K02354 \| \| K08269 \| \| K03099 \| \| K08271 \| \| \| K07208 \| \| --- \| \| K04365 \| \| K03083 \| \| K07205 \| \| K02144 \| \| K03158 \| \| K04467 \| \| K16186 \| \| K02353 \| \| K02353 \| \| K02991 \| \| K02991 \| \| K08266 \| \| K09594 \| \| K09594 \| \| K04364 \| \| K04688 \| \| K03099 \| \| K02833 \| \| K04456 \| \| K07206 \| \| K08272 \| \| K04366 \| \| K03258 \| \| K09594 \| \| K04366 \| \| K13780 \| \| K07207 \| \| K07207 \| \| K16344 \| \| K09594 \| \| K03068 \| \| K04365 \| \| K02353 \| \| K07208 \| \| K01110 \| \| K04688 \| \| K13302 \| \| K04688 \| \| K02353 \| \| K09594 \| \| K07208 \| \| K04456 \| \| K04688 \| \| K07206 \| \| K04366 \| \| K02991 \| \| K03259 \| \| K07209 \| \| K02148 \| \| K02147 \| \| K04688 \| \| K04688 \| \| K04688 \| \| K03068 \| \| K04688 \| \| K06519 \| \| K06519 \| \| K08269 \| \| K06519 \| \| K04456 \| \| K06519 \| \| K06519 \| \| K06519 \| \| K06519 \| \| K06519 \| \| K06519 \| \| K04373 \| \| K06519 \| \| K03258 \| \| K03258 \| \| K03258 \| \| K13780 \| \| K04456 \| \| K04456 \| \| K07827 \| \| K04456 \| \| K08266 \| \| K08266 \| \| K08266 \| \| K07204 \| \| K02353 \| \| K02353 \| \| K02677 \| \| K04688 \| \| K04688 \| \| K04467 \| \| |

| **Table S2 The differentially expressed genes involved in MAPK signaling pathway** | | | |
| --- | --- | --- | --- |
| GeneID | GeneSymbol | Regulation | KO |
| \| ENSG00000100485 \| \| --- \| \| ENSG00000085511 \| \| ENSG00000160691 \| \| ENSG00000115904 \| \| ENSG00000154310 \| \| ENSG00000068305 \| \| ENSG00000154310 \| \| ENSG00000100485 \| \| ENSG00000111816 \| \| \| ENSG00000121068 \| \| --- \| \| ENSG00000138032 \| \| ENSG00000157764 \| \| ENSG00000182247 \| \| ENSG00000068305 \| \| ENSG00000115966 \| \| ENSG00000170142 \| \| ENSG00000177565 \| \| ENSG00000157557 \| \| ENSG00000135341 \| \| ENSG00000177606 \| \| ENSG00000107643 \| \| ENSG00000125845 \| \| ENSG00000115808 \| \| ENSG00000006432 \| \| ENSG00000090372 \| \| ENSG00000055208 \| \| ENSG00000177885 \| \| ENSG00000179295 \| \| ENSG00000134954 \| \| ENSG00000050748 \| \| ENSG00000196470 \| \| ENSG00000078967 \| \| ENSG00000115904 \| \| ENSG00000034152 \| \| ENSG00000164924 \| \| ENSG00000076984 \| \| ENSG00000131508 \| \| ENSG00000138032 \| \| ENSG00000146648 \| \| ENSG00000115966 \| \| ENSG00000082898 \| \| ENSG00000157557 \| \| ENSG00000153721 \| \| ENSG00000138696 \| \| ENSG00000068305 \| \| ENSG00000185386 \| \| ENSG00000112096 \| \| ENSG00000146648 \| \| ENSG00000177565 \| \| ENSG00000107643 \| \| ENSG00000070087 \| \| ENSG00000082898 \| \| ENSG00000107643 \| \| ENSG00000157764 \| \| ENSG00000055208 \| \| ENSG00000177565 \| \| ENSG00000107643 \| \| ENSG00000166913 \| \| ENSG00000153721 \| \| ENSG00000166913 \| \| ENSG00000166913 \| \| ENSG00000082898 \| \| ENSG00000146648 \| \| ENSG00000136238 \| \| ENSG00000113575 \| \| ENSG00000136238 \| \| ENSG00000109332 \| \| ENSG00000109332 \| \| ENSG00000109332 \| \| ENSG00000109332 \| \| ENSG00000113575 \| \| ENSG00000055208 \| \| ENSG00000006432 \| \| ENSG00000006432 \| \| ENSG00000133703 \| \| ENSG00000068305 \| \| ENSG00000068305 \| \| ENSG00000083799 \| \| ENSG00000083799 \| \| ENSG00000090372 \| \| ENSG00000090372 \| \| ENSG00000090372 \| \| ENSG00000090372 \| \| ENSG00000090372 \| \| ENSG00000072401 \| \| | \| SOS2 \| \| --- \| \| MAP3K4 \| \| SHC1 \| \| SOS1 \| \| TNIK \| \| MEF2A \| \| TNIK \| \| SOS2 \| \| FRK \| \| \| TBX2 \| \| --- \| \| PPM1B \| \| BRAF \| \| UBE2E2 \| \| MEF2A \| \| ATF2 \| \| UBE2E1 \| \| TBL1XR1 \| \| ETS2 \| \| MAP3K7 \| \| JUN \| \| MAPK8 \| \| BMP2 \| \| STRN \| \| MAP3K9 \| \| STRN4 \| \| TAB2 \| \| GRB2 \| \| PTPN11 \| \| ETS1 \| \| MAPK9 \| \| SIAH1 \| \| UBE2D4 \| \| SOS1 \| \| MAP2K3 \| \| YWHAZ \| \| MAP2K7 \| \| UBE2D2 \| \| PPM1B \| \| EGFR \| \| ATF2 \| \| XPO1 \| \| ETS2 \| \| CNKSR3 \| \| BMPR1B \| \| MEF2A \| \| MAPK11 \| \| SOD2 \| \| EGFR \| \| TBL1XR1 \| \| MAPK8 \| \| PFN2 \| \| XPO1 \| \| MAPK8 \| \| BRAF \| \| TAB2 \| \| TBL1XR1 \| \| MAPK8 \| \| YWHAB \| \| CNKSR3 \| \| YWHAB \| \| YWHAB \| \| XPO1 \| \| EGFR \| \| RAC1 \| \| PPP2CA \| \| RAC1 \| \| UBE2D3 \| \| UBE2D3 \| \| UBE2D3 \| \| UBE2D3 \| \| PPP2CA \| \| TAB2 \| \| MAP3K9 \| \| MAP3K9 \| \| KRAS \| \| MEF2A \| \| MEF2A \| \| CYLD \| \| CYLD \| \| STRN4 \| \| STRN4 \| \| STRN4 \| \| STRN4 \| \| STRN4 \| \| UBE2D1 \| \| | \| Down \| \| --- \| \| Down \| \| Down \| \| Down \| \| Down \| \| Down \| \| Down \| \| Down \| \| Down \| \| \| Ups \| \| --- \| \| Ups \| \| Ups \| \| Ups \| \| Ups \| \| Ups \| \| Ups \| \| Ups \| \| Ups \| \| Ups \| \| Ups \| \| Ups \| \| Ups \| \| Ups \| \| Ups \| \| Ups \| \| Ups \| \| Ups \| \| Ups \| \| Ups \| \| Ups \| \| Ups \| \| Ups \| \| Ups \| \| Ups \| \| Ups \| \| Ups \| \| Ups \| \| Ups \| \| Ups \| \| Ups \| \| Ups \| \| Ups \| \| Ups \| \| Ups \| \| Ups \| \| Ups \| \| Ups \| \| Ups \| \| Ups \| \| Ups \| \| Ups \| \| Ups \| \| Ups \| \| Ups \| \| Ups \| \| Ups \| \| Ups \| \| Ups \| \| Ups \| \| Ups \| \| Ups \| \| Ups \| \| Ups \| \| Ups \| \| Ups \| \| Ups \| \| Ups \| \| Ups \| \| Ups \| \| Ups \| \| Ups \| \| Ups \| \| Ups \| \| Ups \| \| Ups \| \| Ups \| \| Ups \| \| Ups \| \| Ups \| \| Ups \| \| Ups \| \| Ups \| \| Ups \| \| Ups \| \| Ups \| \| | \| K03099 \| \| --- \| \| K04428 \| \| K06279 \| \| K03099 \| \| K08840 \| \| K09260 \| \| K08840 \| \| K03099 \| \| K08892 \| \| \| K10176 \| \| --- \| \| K04461 \| \| K04365 \| \| K06689 \| \| K09260 \| \| K04450 \| \| K06689 \| \| K04508 \| \| K02678 \| \| K04427 \| \| K04448 \| \| K04440 \| \| K04662 \| \| K17608 \| \| K04417 \| \| K17608 \| \| K04404 \| \| K04364 \| \| K07293 \| \| K02678 \| \| K04440 \| \| K04506 \| \| K06689 \| \| K03099 \| \| K04432 \| \| K16197 \| \| K04431 \| \| K06689 \| \| K04461 \| \| K04361 \| \| K04450 \| \| K14290 \| \| K02678 \| \| K17536 \| \| K13578 \| \| K09260 \| \| K04441 \| \| K04564 \| \| K04361 \| \| K04508 \| \| K04440 \| \| K05759 \| \| K14290 \| \| K04440 \| \| K04365 \| \| K04404 \| \| K04508 \| \| K04440 \| \| K16197 \| \| K17536 \| \| K16197 \| \| K16197 \| \| K14290 \| \| K04361 \| \| K04392 \| \| K04382 \| \| K04392 \| \| K06689 \| \| K06689 \| \| K06689 \| \| K06689 \| \| K04382 \| \| K04404 \| \| K04417 \| \| K04417 \| \| K07827 \| \| K09260 \| \| K09260 \| \| K08601 \| \| K08601 \| \| K17608 \| \| K17608 \| \| K17608 \| \| K17608 \| \| K17608 \| \| K06689 \| \| |
